# Identification and Characterization of an Electric Vehicle’s Operational Regimes Using Sensor Data

**DOI:** 10.3390/s26165085

**Published:** 2026-08-11

**Authors:** Federico Silvestri, Gianluca Canali, Andrea Di Martino, Michela Longo, Paolo Ranieri

**Affiliations:** 1 Department of Energy, Politecnico di Milano, 20133 Milan, Italy; gianluca.canali@polimi.it (G.C.); andrea.dimartino@polimi.it (A.D.M.); 2 School of Medicine and Surgery, Università degli Studi di Milano Bicocca, 20126 Milan, Italy; paolo.ranieri@unimib.it

**Keywords:** internet of things, electric vehicle, integrated mobility, data acquisition

## Abstract

The increasing adoption of electric vehicles (EVs) has led to the generation of large-scale multi-domain datasets containing information on battery, thermal, charging, and driving behavior under real-world operating conditions. These datasets provide valuable insights into vehicle operation by enabling the analysis of interactions among electrical, environmental, and operational variables. This paper presents a comprehensive analysis of a real-world EV dataset collected during on-road operation, focusing on the characterization of the available measurements and their suitability for describing different vehicle operating conditions. The dataset is analyzed through statistical evaluation, correlation analysis, and dimensionality reduction techniques to investigate variable relationships and the representation of vehicle behavior. Specific operating conditions, including driving, charging, regenerative braking, and parking states, are described based on the available sensor measurements and reconstructed information where required. The results highlight the potential and limitations of real-world EV datasets, demonstrating the importance of data quality, variable consistency, and operational state reconstruction for accurately interpreting vehicle behavior and supporting future data-driven mobility applications.

## 1. Introduction

As part of the widespread efforts to reduce GHG emissions, electric vehicles (EVs) have been explored as a solution that is able to contrast life cycle emissions [1,2] with virtuous emissions reduction [3], especially if coupled with electricity’s future carbon intensity reduction [4,5,6]. As reported by the vast literature on the subject, dimensions such as human health and land use are strongly affected by the production of batteries and other components [7], which has led scientists to investigate possibilities such as battery second-life use in storage systems [8], the circular economy [9] and the overall value chain [10]. To evaluate the potential benefits deriving from their implementation, multiple approaches have been developed [11]. Overall, the aims and roles of models in the field vary vastly depending on the specific added value that they are intended to provide. From a systemic point of view, EVs of all types are expected to affect grid stability [12], especially in moments of peak power request [13,14]. In order to contrast these effects, researchers have long studied different solutions, targeting both supply and demand. On the supply side, the location problem for EV charging stations (EVCSs) has been studied both to satisfy users’ demand [15,16,17] and to deliver the optimal redistribution of loads, even in highway contexts [18,19], also employing solutions of virtual power plants (VPPs) and integration with renewable generation [20], as well as more innovative charging solutions, such as wireless power transfer (WPT) [21] and battery swap [22]. Special attention has also been given to specific business models and use cases, such as free floating car sharing [23]. Among the legislative efforts of the European Union, vehicle-to-grid (V2G) and smart cities are frequently investigated in the field [24]. The aforementioned topics are expected to find additional solutions and possibilities in the introduction of intelligent autonomous and connected electric vehicles (IACEVs), which will further enhance the ability of the system to regulate the stability of the grid and the availability of vehicles [25]. On the demand side, dynamic pricing, time-of-use tariffs and smart charging solutions have received increasing attention [26,27] due to their ability to act upstream of the energy distribution, directly targeting the behavioral response of users and, in turn, limiting grid disruptions. By accurately planning and modeling the behavioral [28] side, even positive contributions to stability can be expected [29]. From a vehicular perspective, predictions on the timing of charging sessions [30], comparisons between models and reality [31] and different technologies [32] have all been proven to be reliable tools for planning and operating electric vehicles. In addition to this, models consisting of interactive simulators, digital twins (DTs), machine learning (ML), etc., may still differ in the use cases and solutions provided while partially overlapping in their intents. In this regard, topics of data completeness, reliability, consistency and availability are of primary interest and strongly influence what is achievable from the models. As an introductory example, the reliability of the telemetry affects whether the data can constitute a solid basis on which to build a more complex model, such as a DT, or if the characterization of the variables in the datasets could create inconsistencies in the analysis and therefore weaken the final results. Table 1 proposes a light overview and comparison of the types of models. Research on digital twins (DTs) has demonstrated their potential to enhance several aspects of electric mobility [33,34,35,36]. In this context, this work highlights the importance of characterizing and assessing EV datasets that are intended for advanced data-driven applications, including DT-oriented frameworks. Real-world vehicle operation involves complex interactions between driving behavior, environmental conditions, battery dynamics, and charging processes, which require reliable and consistent data representation. The dataset analyzed in this work is therefore investigated in terms of variable consistency, operational state representation, and information completeness, identifying both its capabilities and limitations. While the development of a DT is outside the scope of this paper, the presented analysis provides relevant insights into the quality and suitability of real-world EV data for supporting accurate digital representations of vehicle operation. In the following analysis, the description of available data is focused on the structure and plausibility of the dataset as provided by the upper part of the data supply chain [37,38]. The aim of this paper is to identify missing values, inconsistencies, and possible discordances arising among sensor measurements, and its main contribution can be found in the methodology of the analysis. To support this, a methodological overview of the methods followed and the associated materials used in the analysis is presented in Section 2, while, in Section 3, the developed results and identified limitations are discussed. In the end, in Section 4, conclusions are drawn and recommendations are proposed.

### Data Source Statement

The dataset used in this study was collected by a voluntary Tesla vehicle owner who consented to the collection and subsequent transfer of the vehicle telemetry data for research purposes. The data were obtained and analyzed with the owner’s permission and in accordance with the applicable legal requirements. Because the dataset contains GPS information that could potentially reveal sensitive locations, including the participant’s residence, the raw dataset is not publicly shared. Access to the data is restricted to the research team. If the dataset is released in the future, all the GPS information and other potentially identifying attributes will be removed to ensure participant anonymity. A formal ethical review was not required because the study involved data that were voluntarily provided by a consenting participant and was conducted in accordance with the applicable institutional policies.

## 2. Materials and Methods

As mentioned in the Introduction, the validation of the digital twin will be conducted starting from the analysis of a real-world dataset, collected over a period of two months between November 2025 and January 2026 for a total time window of 56 days. The dataset was acquired thanks to the Tessie^TM^ Tesla API management platform, which exploits the internal sensors and transducers installed onboard to continuously transmit and collect all the information retrieved during the lifetime of the vehicle 24 h a day, 7 days a week, thus achieving the internet of things (IoT) as applied to EVs [39,40]. In particular, data collection occurs according to two conditions supervised by an event_collector algorithm each 0.5s:

1. Data have changed from the previous value.
2. A time threshold interval_second has elapsed. This value is set differently according to the different transducers being queried.

During real driving operation, a small subset of fields are regularly recorded. This small subset can be enriched according to the preferences of the EV owner:

- Vehicle speed (max 6 data points/min);
- Location (max 6 data points/min);
- State of charge (SoC, 1 data point/min);
- Odometer (1 data point/min);
- Estimated battery range (1 data point/min).

The reduced number of data recorded every minute for each variable is justified by the need to have a light dataset without losing meaningfulness of the quantities recorded. A high-frequency sampling would lead to a heavier dataset to be handled and stored. A short list of common variables being collected is reported in Listing 1, which can be customized by each owner adding other variables.

**Listing 1.** Example of a list of common variables acquired.

“fields”: { 

  “VehicleSpeed”: { “interval_seconds”: 10 }, 

  “Location”: { “interval_seconds”: 10 }, 

  “Soc”: { “interval_seconds”: 60 }, 

  “DoorState”: { “interval_seconds”: 1 }, 

  “Odometer”: { “interval_seconds”: 60 }, 

  “Locked”: { “interval_seconds”: 1 }, 

  “EstBatteryRange”: { “interval_seconds”: 60 }, 

  “ChargeAmps”: { “interval_seconds”: 1 }, 

  “DetailedChargeState”: { “interval_seconds”: 1 }, 

  “VehicleName”: { “interval_seconds”: 1 }, 

  “TpmsPressureFl”: { “interval_seconds”: 1 }, 

  “TpmsPressureFr”: { “interval_seconds”: 1 }, 

  “TpmsPressureRl”: { “interval_seconds”: 1 }, 

  “TpmsPressureRr”: { “interval_seconds”: 1 }, 

  “TpmsLastSeenPressureTimeFl”: { “interval_seconds”: 1 }, 

  “TpmsLastSeenPressureTimeFr”: { “interval_seconds”: 1 }, 

  “TpmsLastSeenPressureTimeRl”: { “interval_seconds”: 1 }, 

  “TpmsLastSeenPressureTimeRr”: { “interval_seconds”: 1 } 

} 



The vehicle considered is a Tesla Model 3 Basic Model Standard Range (Plus) for private use displaced in northern Italy, with the majority of trips being located in Lombardy and some exceptional journeys in the Piedmont region. The EV had already been in service for several years, accumulating almost 35,000 km and presenting fully charged energy of 58.2 kWh. Further information can be found in Table 2.

The models will be presented in the following Section 2.1, with the consequent exploratory data analysis in Section 2.2.

### 2.1. Methodological Overview of the Analysis

To build the digital twin and the associated validation platform, quality of the data must be ensured. To achieve this goal, the analysis has been structured as follows:

1. Computation of summary statistics of the dataset;
2. Generation of visualizations for the longitudinal data;
3. Check of coherence between reported information;
4. Analysis of criticalities that emerged from the study;
5. Understanding of the implicit assumptions and associated consequences.

This approach can be justified by considering the complexity of the data and the consequent multi-step iterative understanding of the limitations of the collected data. Summary statistics can be used to understand the structure of a dataset. Average values, with associated standard deviations, can be used as a first proxy of the expected value of the whole dataset, offering a first step of dataset validation when confronted with the underlying real-world scenario. Furthermore, the collected dataset is constructed as a succession of batches of data due to the intrinsic seasonality of human driving behavior. Despite this, irregular behavior can be expected, with strongly different driving ranges as well as battery states, leading to different charging approaches and timings. The driving states can, in general, vary strongly for a number of factors, such as traffic, type of road or other vehicle-related factors, but also fatigue of the driver and meteorological conditions. In the direction of automatization of the process with low to minimal human intervention, more attention will be given to what can be inferred from the dataset, avoiding cross-referencing with routing services or other road-related physical factors.

As explained in the later sections of this work, during the research, the need to define different regimes naturally arose from the data. Specifically, five regimes are introduced and defined: baseline, weak, weak clean, strict and regenerative. Their definitions were developed according to progressively more restrictive criteria, with the objective of distinguishing plug-in charging events, regenerative braking phases, and standard vehicle operation in a robust and hierarchical manner.

The weak charging regime identifies any observation for which at least one charger-related variable indicates non-zero charging activity. In the following, $P_{c}$, $I_{c}$, and $V_{c}$ denote the charger power, charger current, and charger voltage, respectively.

(1) $$C_{\mathrm{weak}}=\left\{x:P_{c}>0\;\vee \;I_{c}>0\;\vee \;V_{c}>0\right\}$$

**Figure 1:**
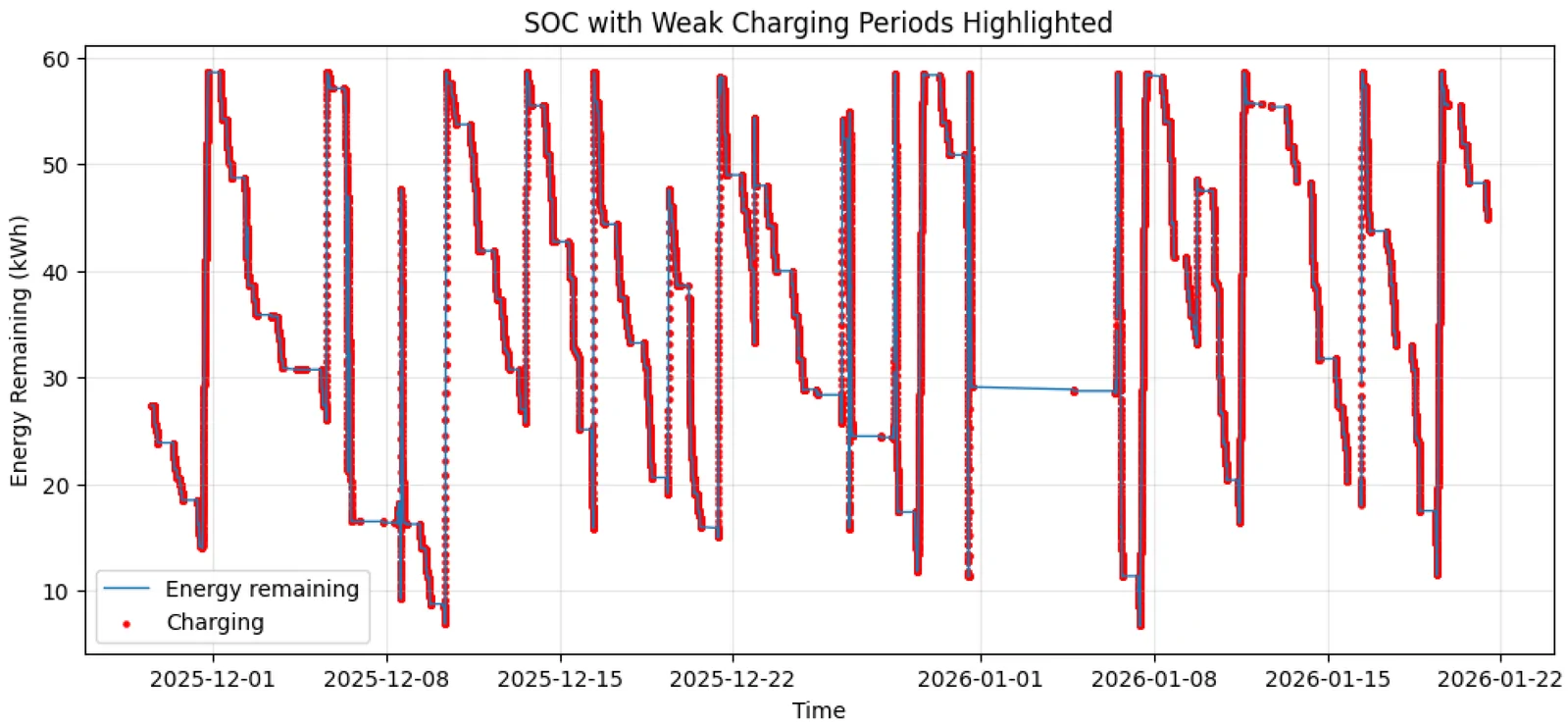
Plot of the evolution of the energy remaining in the battery for the weak identification.

This definition is intentionally permissive as it is designed to capture all observations exhibiting any measurable indication of charging activity independently of its magnitude or physical consistency. A first-attempt classification according to this criterion is depicted in Figure 1.

To mitigate the influence of measurement noise and isolated low-amplitude fluctuations, a refined definition of weak was introduced, named weak clean. An observation is classified as weak clean charging if at least one charger-related variable exceeds empirically defined thresholds:

(2) $$C_{\mathrm{weak}\_\mathrm{clean}}=\left\{x:P_{c}>1\;\mathrm{kW}\;\vee \;I_{c}>1\;A\;\vee \;V_{c}>3\;V\right\}$$

**Figure 2:**
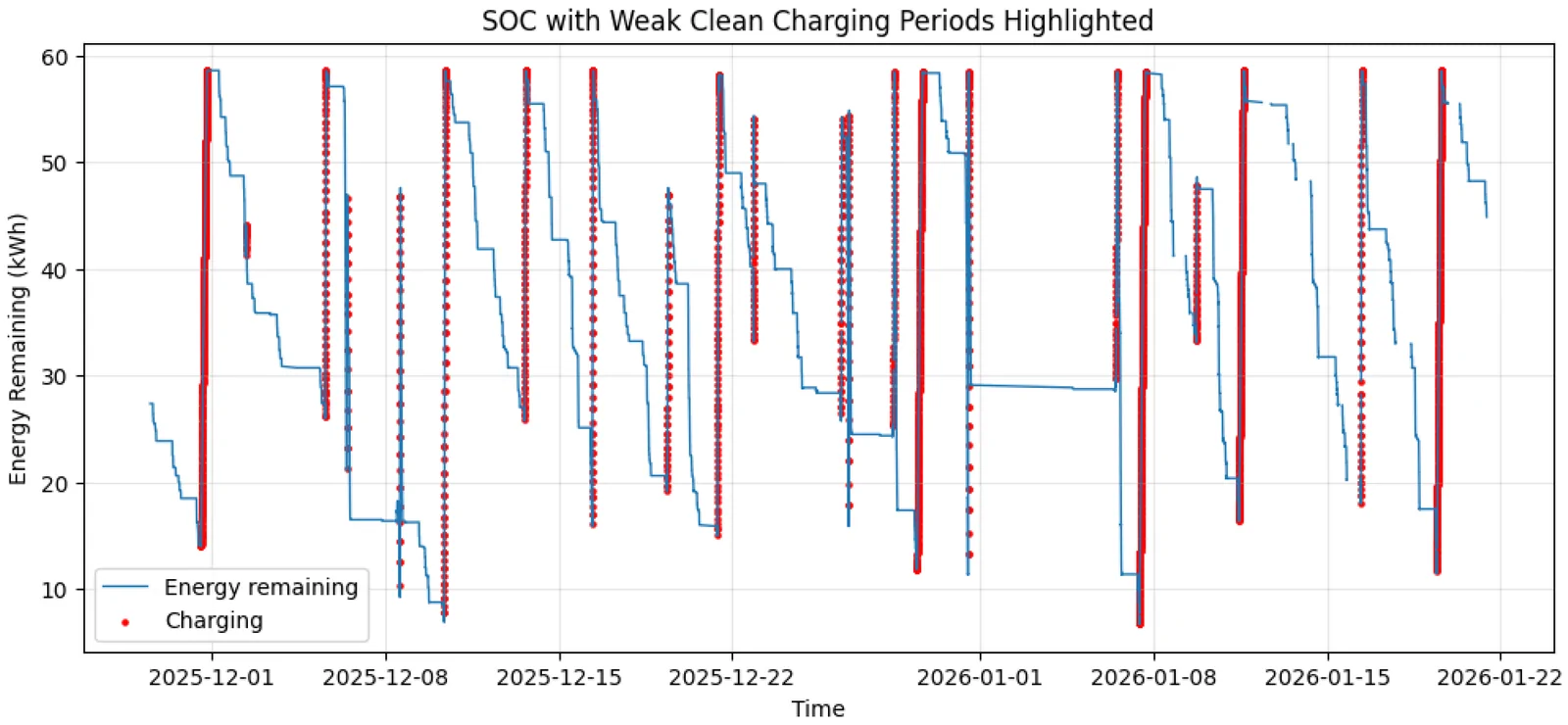
Plot of the evolution of the energy remaining in the battery for the weak cleaned identification.

The selected thresholds were chosen to suppress spurious low-level signals while preserving charging events. As a result, this formulation provides a more robust representation of charging activity compared to the purely non-zero criterion. The change in charging classification is prompted out in Figure 2.

Given the extreme number of states that are identified by the weak mask, it has been discarded, and, in the following, the weak clean mask will be referred to as weak for simplicity. A more conservative criterion was adopted to identify unequivocal charging events, named strict. An observation is classified as strict charging only when all charger-related variables simultaneously indicate active charging:

(3) $$C_{\mathrm{strict}}=\left\{x:P_{c}>0\;\wedge \;I_{c}>0\;\wedge \;V_{c}>0\right\}$$

**Figure 3:**
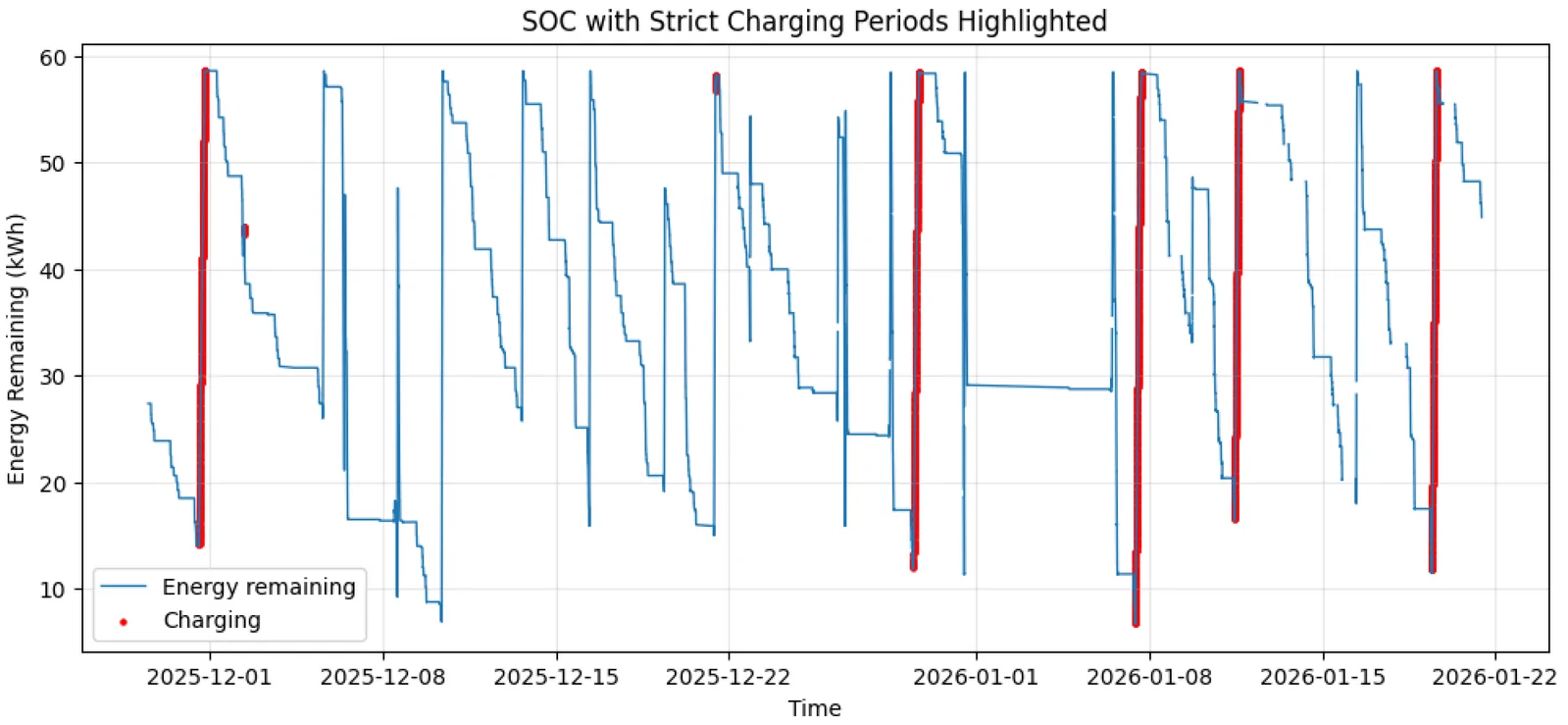
Plot of the evolution of the energy remaining in the battery for the strict identification.

The requirement for concurrent positive power, current, and voltage significantly reduces the probability of false positives arising from isolated sensor noise or transient operating conditions, as recalled by Figure 3.

The regenerative braking regime is defined as

(4) $$R=\{x:S=D\wedge v>0\wedge I_{\mathrm{pack}}>0\wedge P_{c}=0\wedge a<0\wedge \neg W_{c}\}$$

where *S* denotes the transmission shift state, *v* the vehicle speed, $I_{\mathrm{pack}}$ the battery pack current, $P_{c}$ the charger power, *a* the longitudinal acceleration, and $W_{c}$ the weak (clean) charging regime.

**Figure 4:**
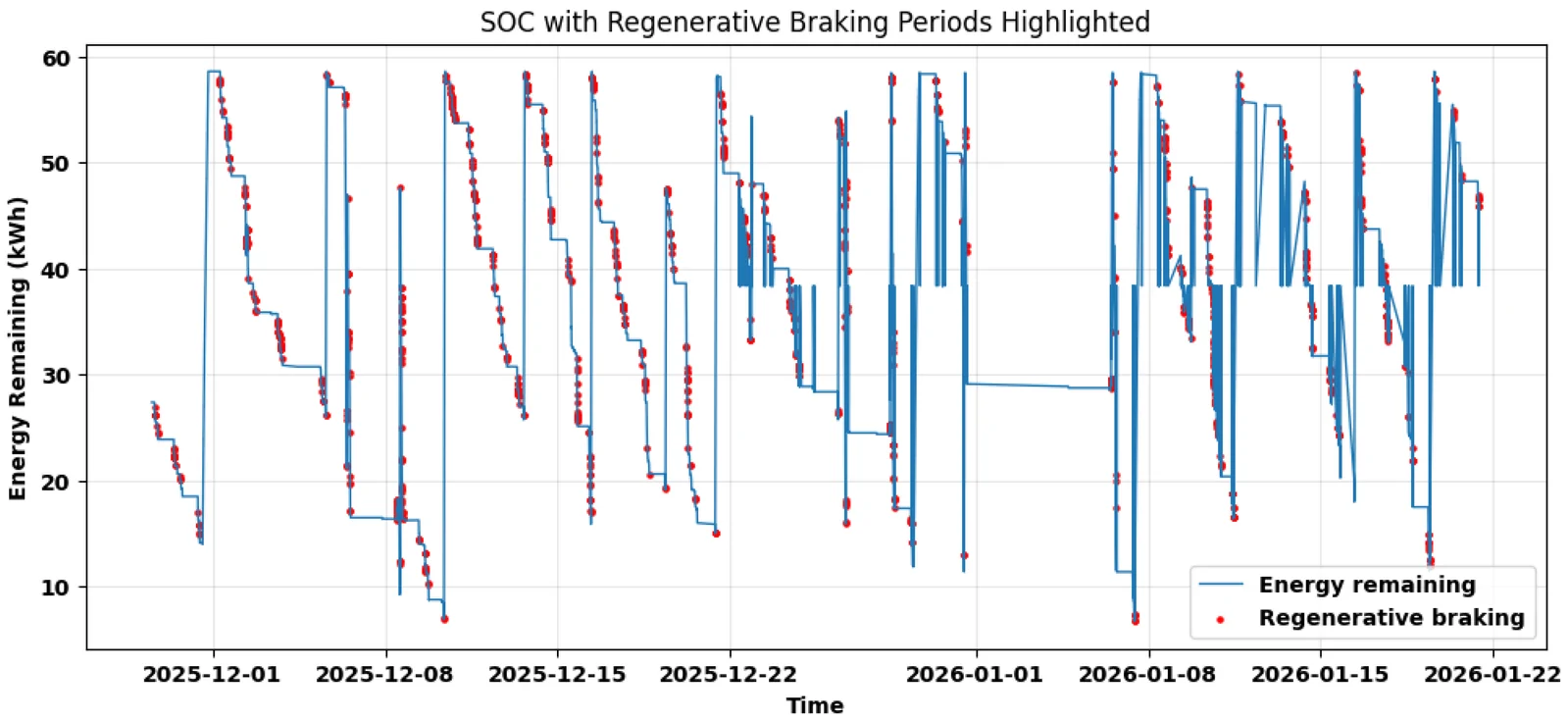
Plot of the evolution of the energy remaining in the battery for the regenerative identification.

This definition identifies regenerative braking events as operating conditions in which the vehicle is in drive mode, moving with positive velocity, and exhibiting positive battery pack current while no external charging power is detected. The additional constraint of negative acceleration (a<0) ensures that only deceleration phases are considered, distinguishing regenerative energy recovery from other positive-current conditions occurring during vehicle operation as Figure 4 depicts. Furthermore, exclusion of the weak charging regime prevents low-power or ambiguous charging events from being misclassified as regenerative braking. Under these conditions, the measured increase in battery energy is attributed to kinetic energy recovery through regenerative braking.

In the end, the baseline operating regime is defined as the complement of charging and regenerative braking conditions:

(5) $$B=\Omega \setminus \left(C_{\mathrm{weak}\_\mathrm{clean}}\cup R\right)$$

This regime represents standard vehicle operation and serves as the reference condition against which both charging behavior and regenerative energy recovery are evaluated.

Among the tools used in the analysis, apart from longitudinal plotting, principal component analysis had a foundational role. Principal component analysis (PCA), after a first step of data standardization, works by generating a rotation of the axes in the direction that maximizes the variance and has long been used in the analysis of data for its explanatory capabilities [41,42,43,44]. The importance of this role can be attributed to the ability to provide a *k*-dimensional visualization of the dataset depending on the accepted threshold of mathematical explanatory ability associated. As mentioned earlier in this subsection, the strong alternations of clusters of driving and stationary states generate batches of semi-continuously evolving data, meaning that, in the *k*-dimensional visualization, some degree of continuity can be expected. Other visualization techniques have been considered and used in the work, such as uniform manifold approximation and projection for dimension reduction (UMAP) [45], which is better suited for preserving local structures than PCA. In the analysis, PCA will be used for system evolution and identification, while UMAP is less interpretable as a visual check of coherence of the information. As mentioned, different masks have been introduced to evaluate the identificatory ability of the registered states of the vehicle, and metrics have been computed to give an associated mathematical interpretation of the findings. As an example, a behavioral regime *g* can be defined as a set of points ${\left\{x_{i}\right\}}_{i=1}^{n_{g}}\subset R^{d}$, where each $x_{i}$ represents an observation in the feature space. The centroid of regime *g* is defined as

$$\mu_{g}=\frac{1}{n_{g}}\sum_{i=1}^{n_{g}}x_{i},$$

while the global centroid over all observations is:

$$\mu =\frac{1}{N}\sum_{i=1}^{N}x_{i}.$$

The compactness of a regime is then defined as the within-cluster variance:

$$C_{g}=\frac{1}{n_{g}}\sum_{i=1}^{n_{g}}{∥x_{i}-\mu_{g}∥}^{2}.$$

Low values of $C_{g}$ indicate a tightly concentrated behavioral regime, while higher values indicate heterogeneous or dispersed behavior.

The centrality of a regime is defined as the squared distance between the regime centroid and the global centroid:

$$Z_{g}={∥\mu_{g}-\mu ∥}^{2}.$$

Low centrality corresponds to regimes that lie close to the global behavior, whereas high centrality indicates regimes that represent specialized operational modes, such as charging. The dispersion of a regime is defined as the average pairwise distance between points:

$$D_{g}=\frac{1}{n_{g}\left(n_{g}-1\right)}\sum_{i\neq j}∥x_{i}-x_{j}∥.$$

This measure captures the spread and structural coherence of the regime, where low values indicate more compact modes and high values indicate more fragmented or elongated structures.

The Gini index of a regime is computed over the distances of points to the cluster centroid. Let:

$$r_{i}=∥x_{i}-\mu_{g}∥.$$

Then the Gini index is defined as:

$$G_{g}=\frac{\sum_{i=1}^{n_{g}}\sum_{j=1}^{n_{g}}\left|r_{i}-r_{j}\right|}{2n_{g}^{2}\bar{r}},\;\mathrm{where}\;\bar{r}=\frac{1}{n_{g}}\sum_{i=1}^{n_{g}}r_{i}.$$

A low Gini index indicates homogeneous behavior within the regime, while a high Gini index indicates that a small number of extreme events dominate the internal structure of the cluster. Collectively, these metrics can be used to define a characterization of operational regimes, where compactness captures internal cohesion, centrality expresses deviation from global behavior, dispersion describes structural spread, and the Gini index measures internal heterogeneity driven by rare but significant events.

### 2.2. Exploratory Data Analysis

The original dataset retrieved is composed of 9 CSV files, describing states pertaining to:

- The battery;
- Charging procedures;
- Internal and external thermal conditions (air conditioning system);
- Driving and vehicle phases;
- Tire pressure.

The dataset consisted of 26 non-null variables originating from multiple vehicle telemetry streams characterized by different levels of missingness. Timestamp synchronization was performed by constructing a unified master timestamp index as the union of all timestamps available across the individual data sources rather than selecting one sensor as the temporal reference. This approach avoided introducing a bias toward a specific data source while preserving all the available measurements. Prior to synchronization, duplicate timestamps within each data stream were removed. Duplicate removal affected a maximum of 86 samples per single dataset (0.323% of the corresponding records), with 38 duplicates removed from driving states (0.277%) and no duplicates removed from tire pressure measurements. After synchronization, the resulting unified dataset contained 26,947 timestamped rows.

Missing values were retained as NaN to preserve the original measurement availability and avoid introducing artificial values through interpolation. The distribution of missing data across variable groups is summarized in Table 3.

**Figure 5:**
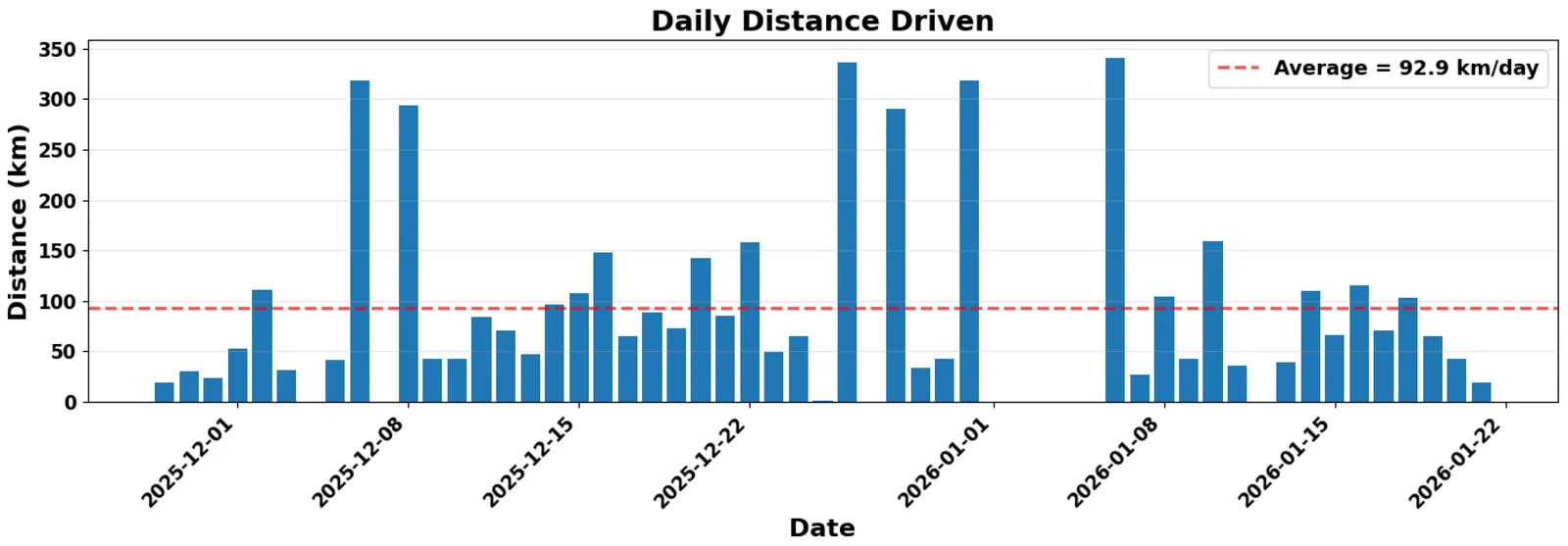
Daily mileage of the vehicle; in red, average overall value.

The synchronized dataset included periods of both vehicle operation and inactivity, as testified by Figure 5. During the observation period, daily traveled distances exceeded 300 km on peak days, with an average traveled distance of approximately 93 km/day.

**Figure 6:**
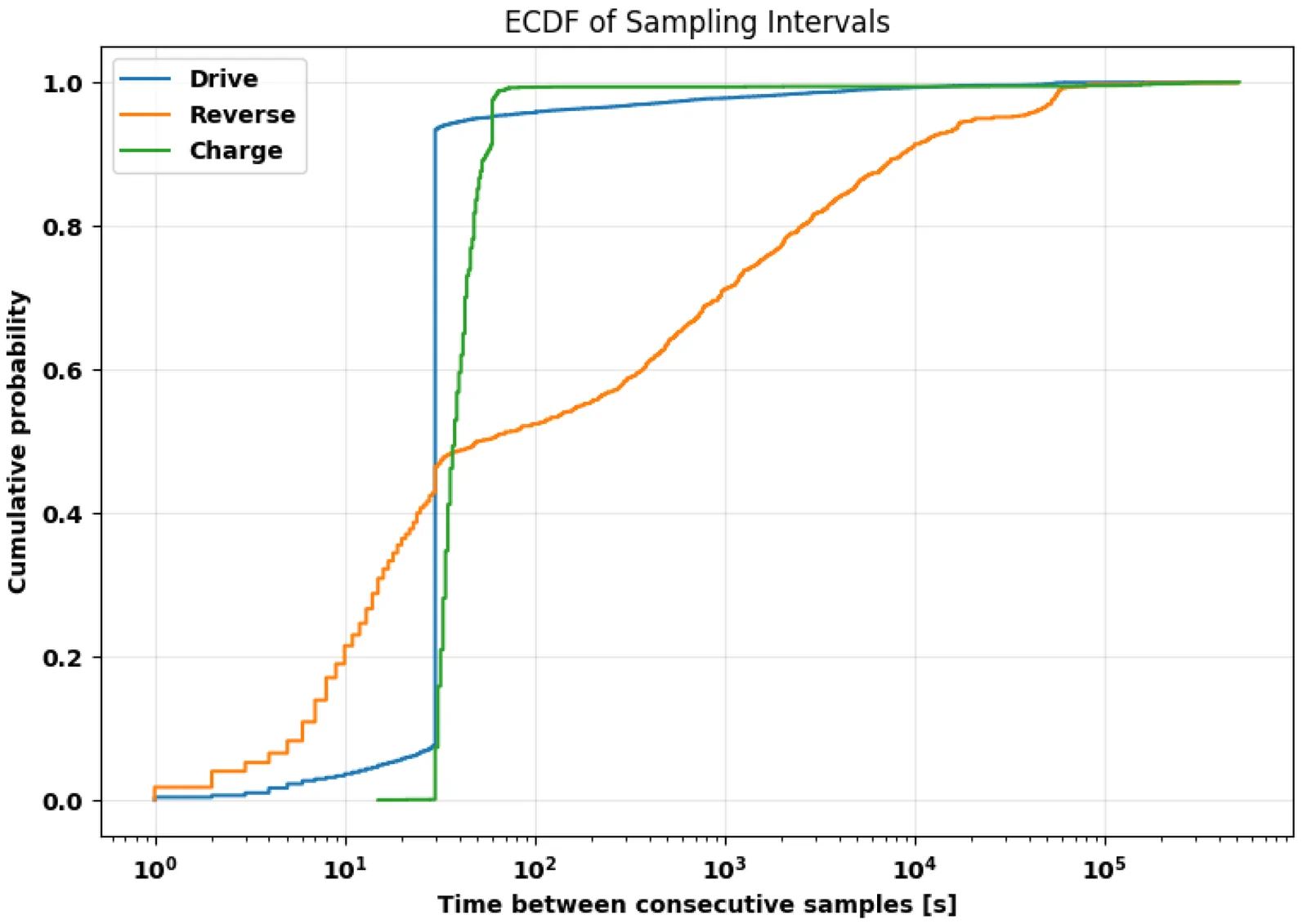
ECD plot of the time between instances of data, divided by charge, drive and reverse states.

The sampling frequency was strongly varying and influenced by the state of the categorical variables. To help visualize the heterogeneity in the time passing between two successive points an empirical cumulative density plot has been provided Figure 6, representing on the *x*-axis the delta in time and on the *y*-axis the share of the dataset collected at least at that delta.

**Figure 7:**
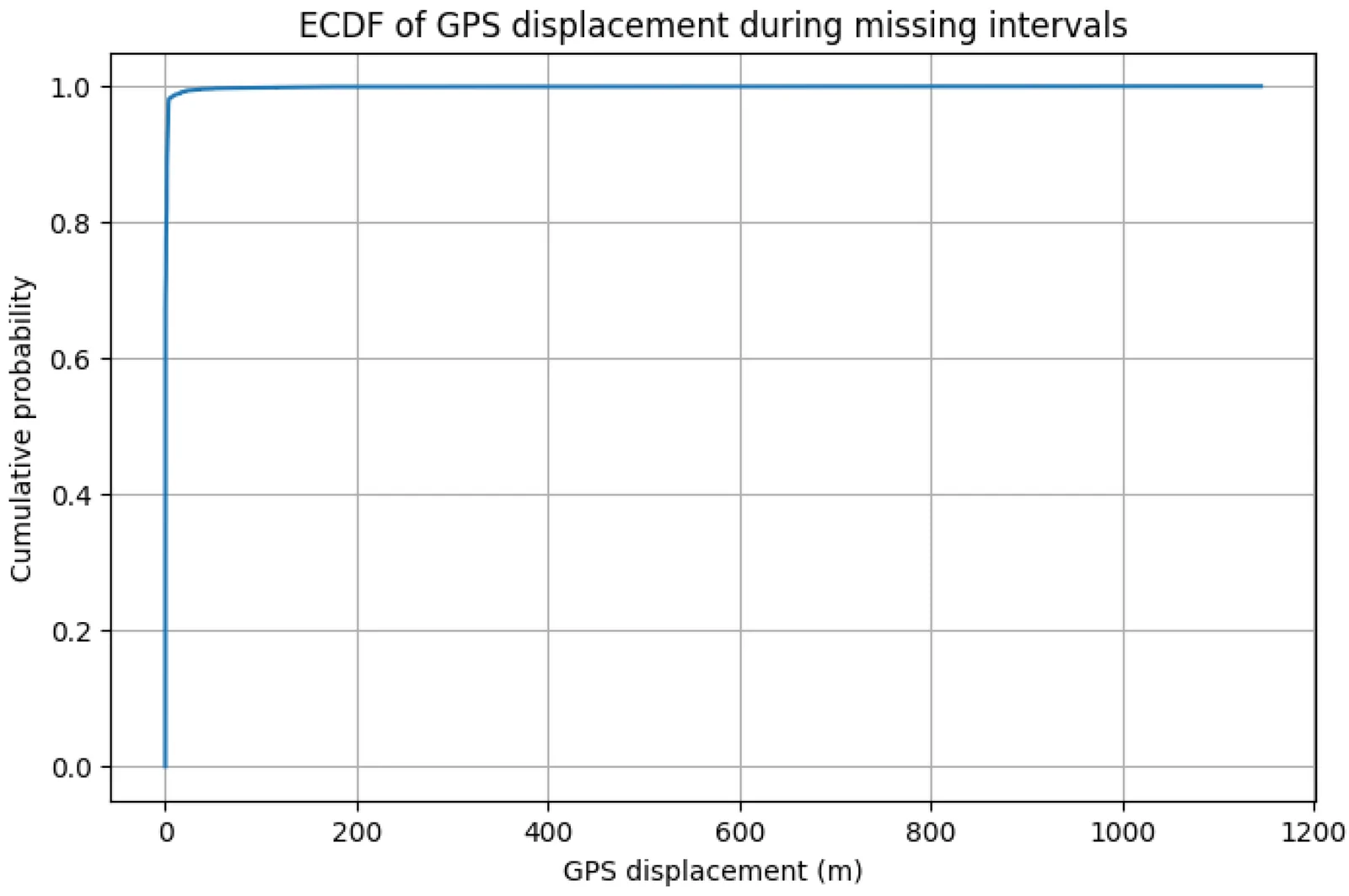
ECDF of the distribution of distances between missing GPS values, as obtained by forward and backward filling the missing values.

One of the main challenges encountered during the analysis was the presence of missing values in the shift state variable, together with several other variables originating from the same data source. Since these signals are essential for distinguishing vehicle operating regimes, the missing observations had to be reconstructed before the subsequent analyses could be performed. By cross-referencing the values of ‘locked’ and ‘sentry mode’ and by considering the difference in positions between forward filling and backward filling the missing values, the authors decided to assign to all the missing values pertaining to the ‘shift state’ the value of ‘reconstructed p’. As an example, in Figure 7, the ECDF of the distribution of distances between forward filling and backward filling is presented. The assignment of the reconstructed parking label is further supported by a manual inspection of the corresponding GPS locations, which consistently corresponded to locations where the vehicle was typically parked. After reconstructing the shift state variable, the remaining missing telemetry variables were imputed using a state-specific approach: for each shift state, the median value of each variable was computed and used to replace missing observations. This strategy preserves the typical operating characteristics associated with each vehicle state while reducing the influence of outliers. Table 4 highlights the distinct electrical behavior associated with the three analyzed shift states.

As expected, the battery state of charge remains remarkably stable across all the regimes, with approximately 37 kWh of the remaining energy and an average usable battery level close to 62%. Similarly, the pack voltage varies only marginally (353–359 V), reflecting the fact that battery voltage is primarily governed by the state of charge rather than the instantaneous operating mode. The largest differences emerge in the current and charging-related variables. During drive, the average pack current is strongly negative (−20.08±45.72 A), indicating that electrical energy is predominantly drawn from the battery to propel the vehicle. Consistently, charger current, charger voltage and charger power are essentially zero, confirming the absence of external charging while the vehicle is in motion. The parking state seems to represent stationary periods in which the vehicle is parked but not actively connected to a charger. This is reflected by charger current and charger power remaining effectively zero (0.00±0.00 A and 0.04±1.34 kW, respectively). The pack current is also substantially closer to zero (−6.74±26.78 A), suggesting only low-power auxiliary electrical loads while the vehicle is idle. In contrast, the reconstructed parking state exhibits a markedly different electrical characterization. The positive average pack current (14.37±42.11 A), together with substantially larger charger voltage (44.11±88.45 V), charger current (2.59±5.68 A) and charger power (6.00±16.04 kW), clearly indicates that this reconstructed regime captures periods during which the vehicle is stationary and externally connected to a charging source. These observations provide quantitative validation of the reconstruction procedure: although GPS information was unavailable for these intervals, the electrical variables consistently identify them as charging events rather than simply parked states. Overall, the reconstructed parking state is therefore not merely an interpolation of missing shift state values but corresponds to a physically distinct operational regime characterized by active battery charging. Conversely, the native parking state captures stationary periods without external energy transfer, while the driving state is dominated by battery discharge associated with vehicle propulsion. The separation between these regimes demonstrates that the reconstructed label preserves meaningful operational information and is supported by independent measurements from the vehicle’s electrical system.

**Figure 8:**
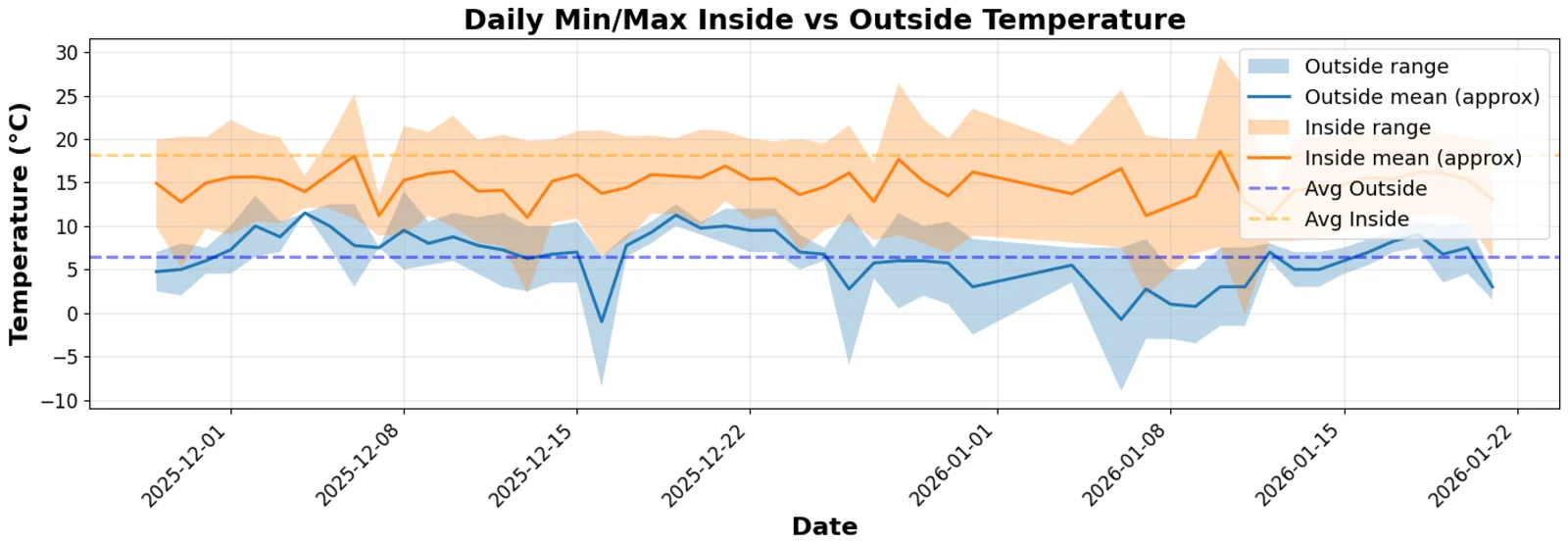
Time-plot of the evolution of daily internal and external temperatures in the considered period.

Average recorded temperatures were below 10 °C, with peaks of −10 °C outside and almost 30 °C inside the vehicle, representative of the stressful working conditions of the heating system and, in turn, battery, as visible in Figure 8. Such working conditions have been vastly identified to be critical for electric vehicles of all sorts [46,47], even requiring preheating procedures for operating the vehicle [48].

**Figure 9:**
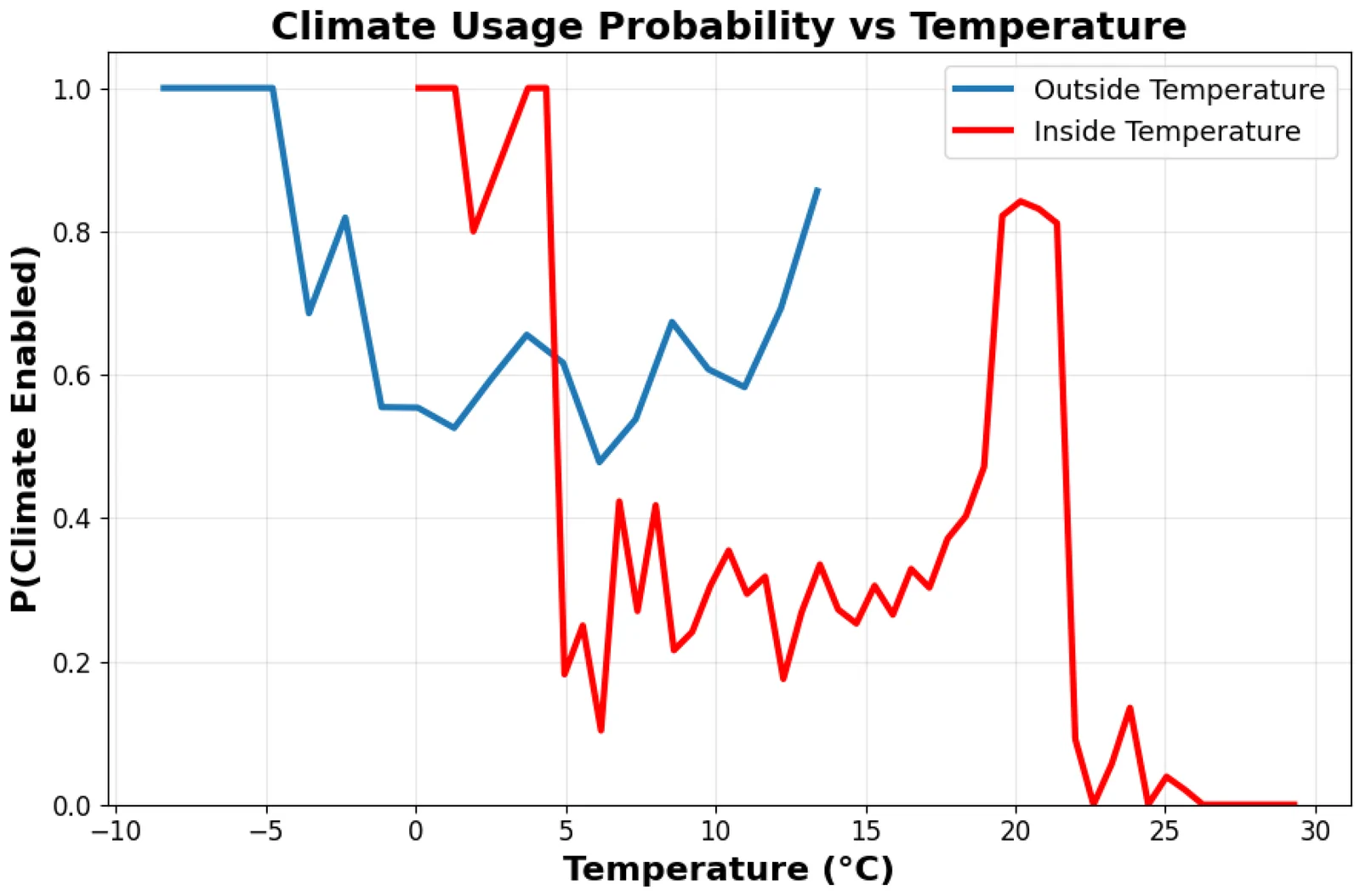
Empirical conditional probability curve of the relationship between activation of AC and outside and inside temperatures.

Additionally, an extraordinary peak of −20 °C has been recorded, although georeferenced local temperatures never dropped below 0 °C, most likely indicating a misreading by the sensors. In the direction of avoiding further manually referencing external sources on a frequent basis, the implemented measure was to define bounding box conditions for the territory and setting outliers to missing values. The defined box consists of the range [−15,50], which can be considered largely conservative. In the end, only four values were set to NaN, all originally below −15 °C. Considering the average outside temperature being below 10 °C, it is expected to have an influence on the activation of air conditioning, as visible in Figure 9, where the probability of having the AC is plotted against outside and inside temperatures.

**Figure 10:**
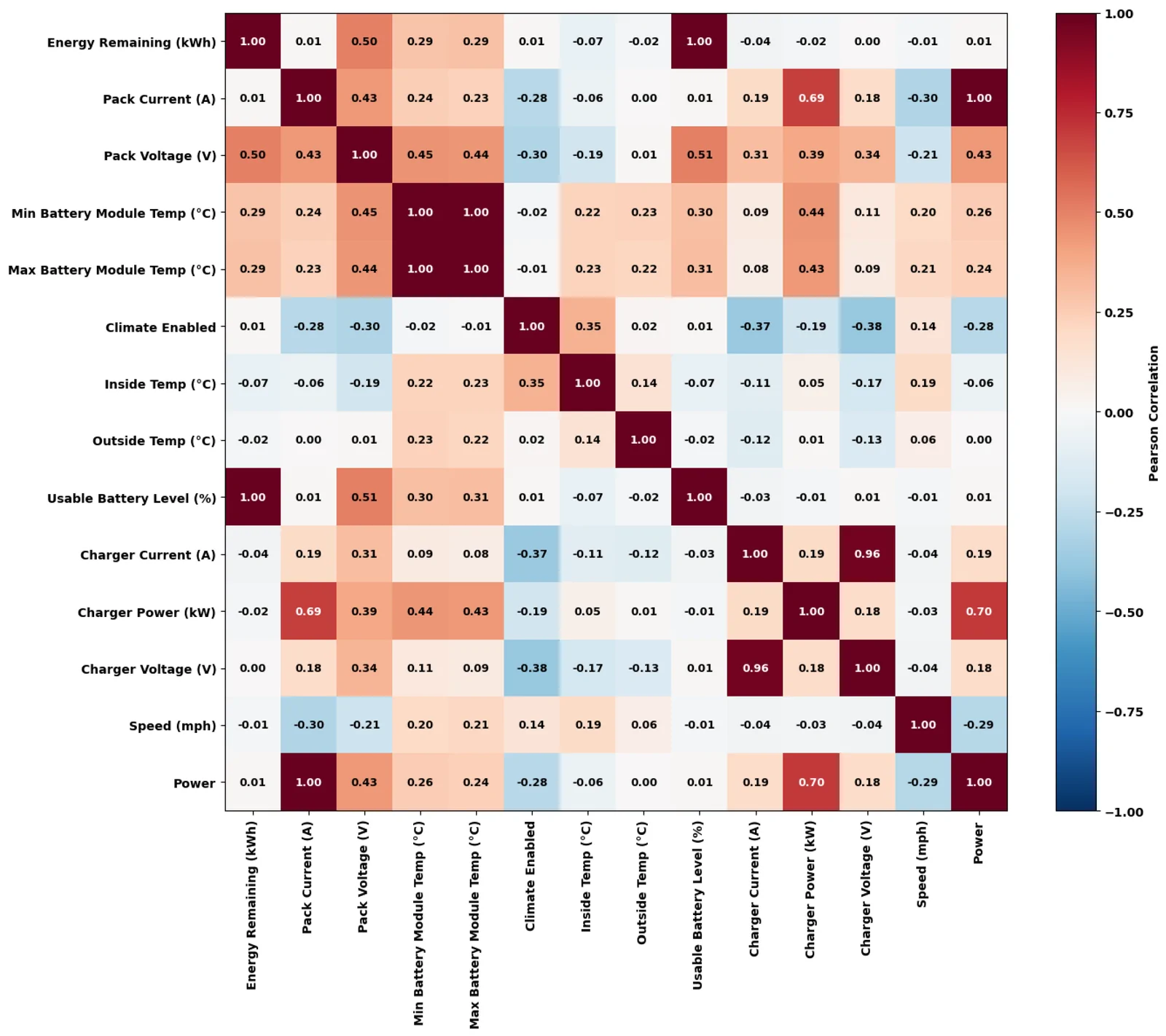
Pearson’s correlation for all numerical variables and encoded categorical variable ‘climate_enabled’.

While the relationship between outside temperature and enabling of AC depends on the actual state of use of the vehicle, the peak at 20 °C for the inside temperature probably indicates the set internal temperature from the driver. To show the association between variables, Pearson’s correlation has been computed for the numerical variables as in Figure 10. The description is intended as an overall insight into the general dynamics of the dataset since the more complex dynamics of state-based and time-evolving values call for a more in-depth analysis. To further support the readers without breaking the flow of this section, in Appendix A, regime-based Pearson’s correlations are proposed. Here and in the following, all the variables and associated numerical considerations will be kept in native logger units, meaning, as an example, that speed will be kept in mph and range in miles. Of particular interest, as will be explained in the later sections, the correlation between ‘charger power’ and ‘pack current’ stands out with one of the highest correlations at 0.69.

**Figure 11:**
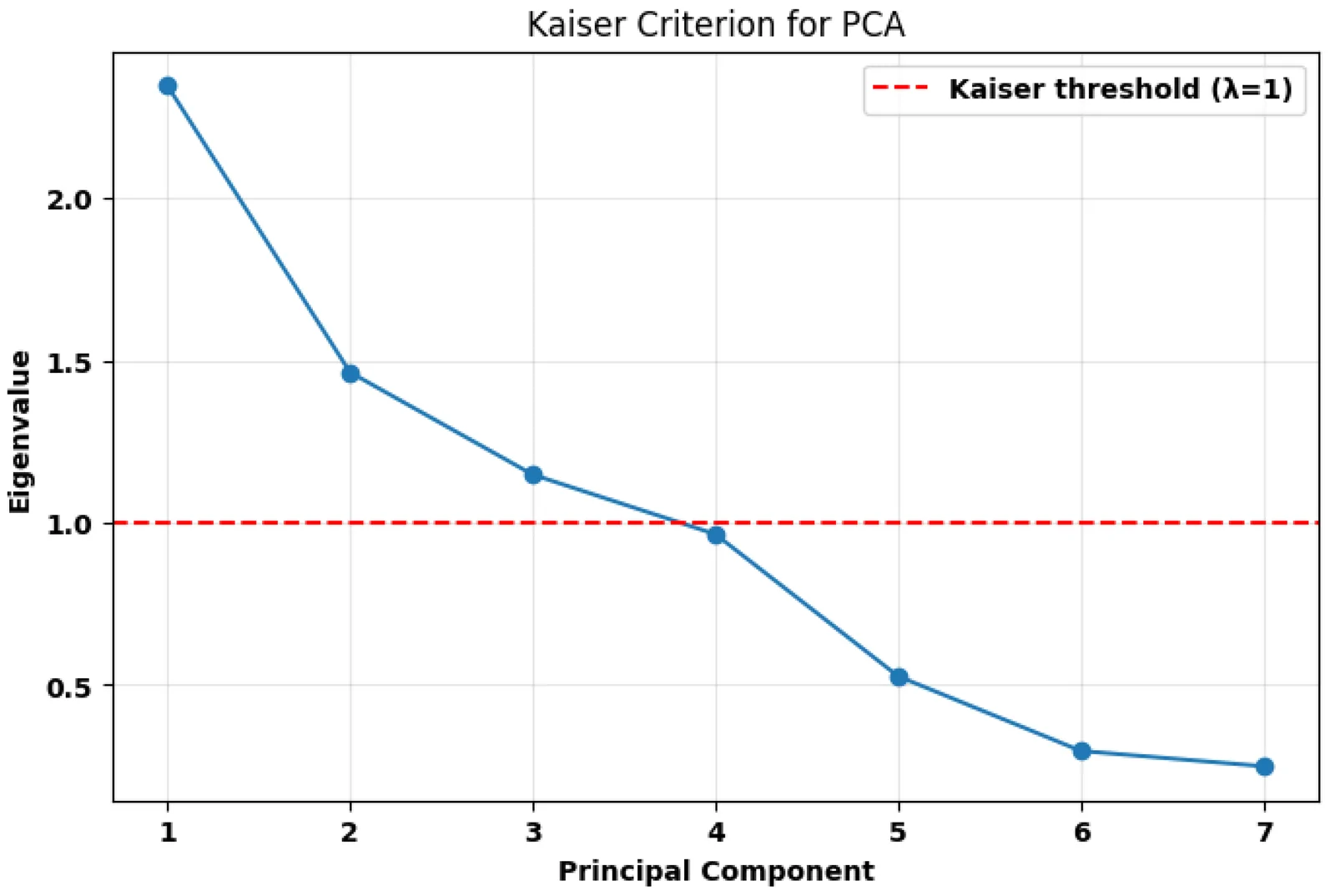
Kaiser plot for choice of dimensionality reduction through PCA showing the number of PCs versus the associated eigenvalue.

On the opposite side, ambient temperatures do not seem to correlate with other variables except for values in the order of 0.2 for the temperatures of the batteries. As a first step in the exploratory analysis of the dataset, a principal component analysis (PCA) has been developed starting from a subset of the variables, avoiding highly repetitive information (such as odometer or lifetime energy used). To support the choice of variables, variance inflation factor (VIF) has been iteratively computed, with the results shown in Table 5. Although the values associated with pack voltage and energy remaining can still be considered rather high, they have both been kept in order to better represent the operational conditions of the vehicle. However, with the analysis proposed in this paper being purely descriptive, the risk of multicollinearity is again attenuated due to the absence of predictive modeling. Together, the first two principal components explain above 50% of the variance (specifically 33.54% for PC1 and 20.91% for PC2). The choice of the number of PCs was 3 based on the Kaiser criterion, with the fourth presenting an eigenvalue below 1 and thus being discarded, as visible in Figure 11.

Although in this first part of the analysis only the results relative to the first two PCs will be presented, later in the paper, the distribution related to the third one will also be shown. The technical aspects about the PCA can be found in Table 6 and Table 7.

**Figure 12:**
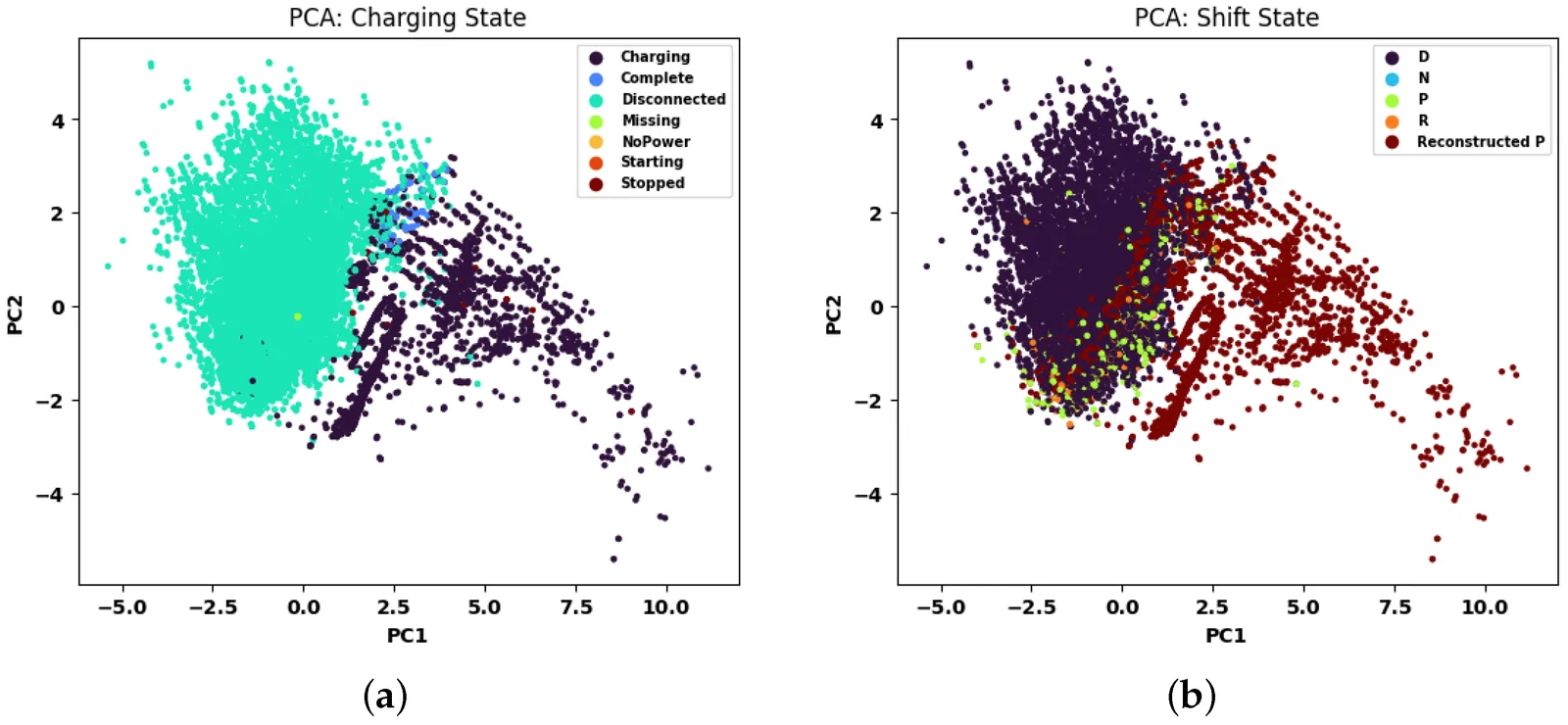
PCA results characterized by (**a**) state of charging and (**b**) state of traction.

To start the analysis, the overall state of the system shows behaviors characterized by both the charging state (Figure 12a) and the shift state Figure 12b. In the charging state visualization it has been needed to assign the ‘missing’ label to all the points pertaining to states where the categorical information was registered for only a subset of the regimes, while, thanks to the reconstruction of the label of ‘reconstructed p’ previously presented, this need was absent for shift states.

**Figure 13:**
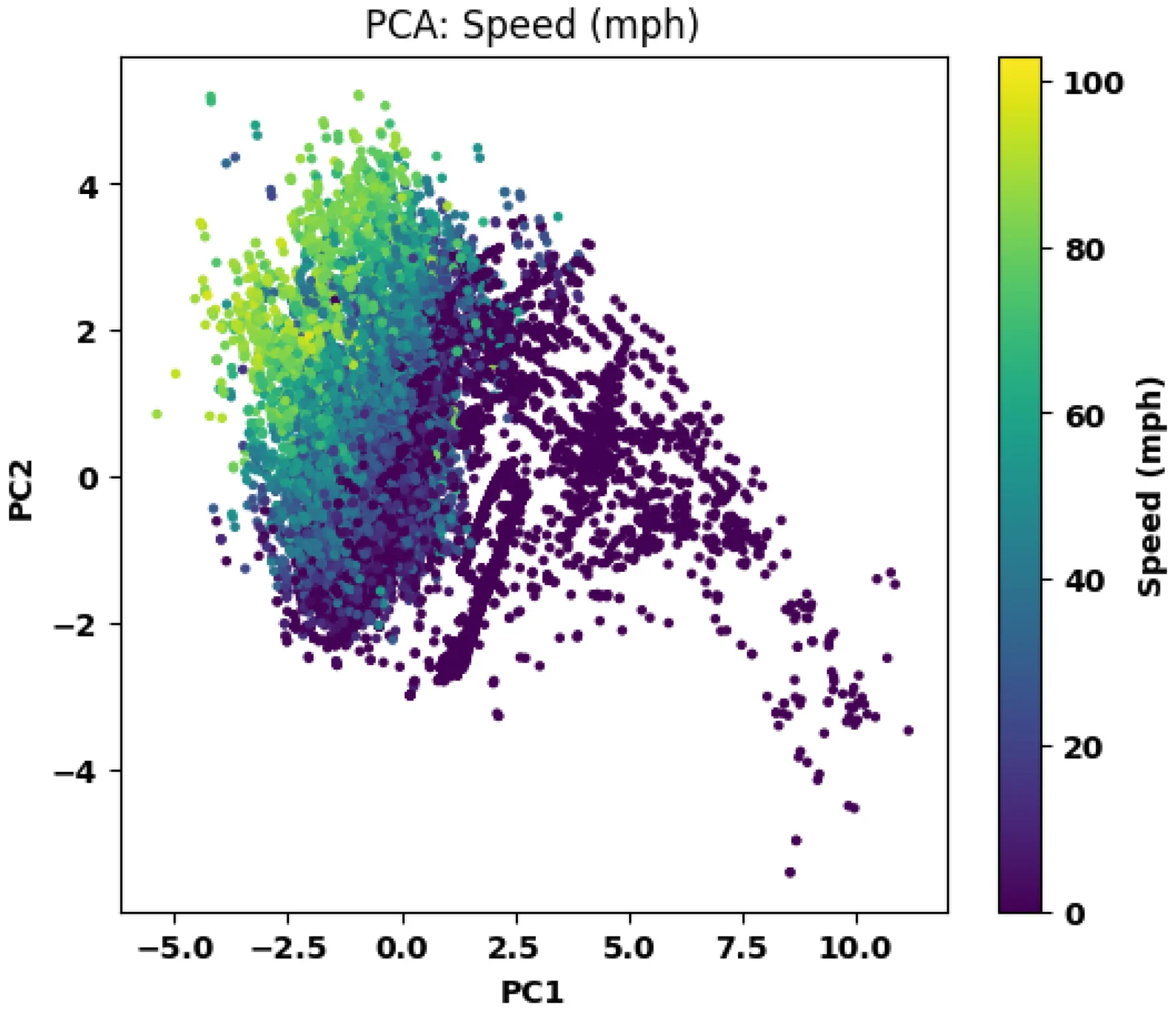
PCA results characterized by speed of the vehicle.

Interestingly, the various regimes appear to be strongly separated in the PCA state representations and seem to show two complementary behaviors. As for the charging state, there is an abundance of disconnected states, with a rather high dispersion in the right side of the representation, possibly indicating variations also inside similarly labeled points. On the other hand, the shift states seem to have been registered in most cases only when driving, possibly indicating absence of registration when the vehicle was turned off or stationary. Furthermore, actively registered parking states ‘P’ seem to be located in the transition zone between the actual driving and reconstructed parking values. Based on these results, the analysis focused on understanding the influence of these parameters, separately addressing the shift state and the charging state, as well as the relations between such categories. As for the shift state, by further characterizing the principal component representation based on the speed of each point, it is possible to visualize the convergence of the system to a rather dense region of higher speeds (Figure 13).

**Figure 14:**
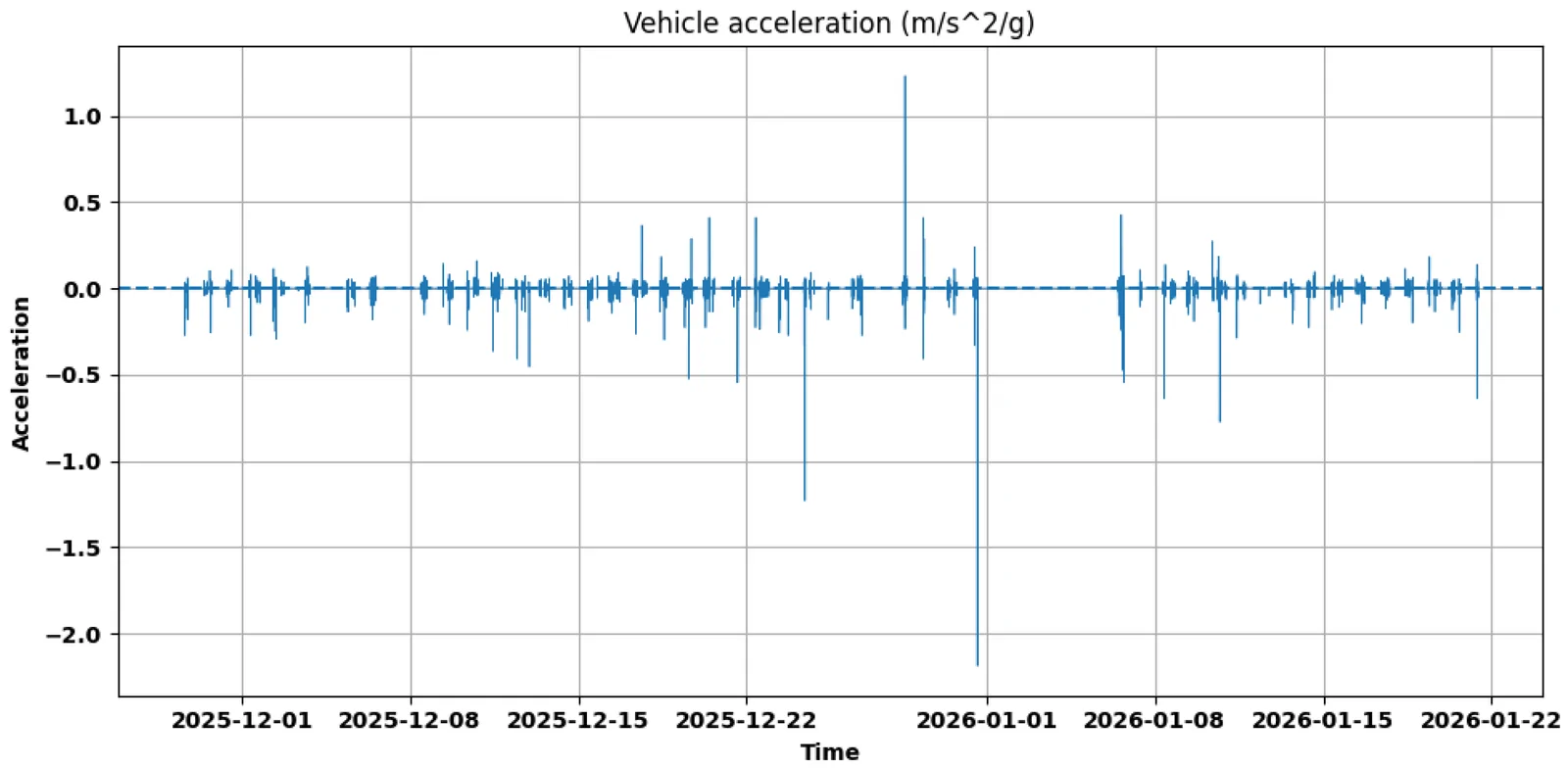
Plot of the time series of acceleration, provided as ratio over g.

Consistent with the information presented in the previous Figure 12b, an overlap between driving shift states and speed can be found. Considering the results shown in Figure 13, the next natural step was to analyze the associated accelerations. In general, the value had to be computed since it was not directly provided by the datasets and was then computed as the variation in recorded speed over the time between two successive timestamps, as shown in Figure 14.

**Figure 15:**
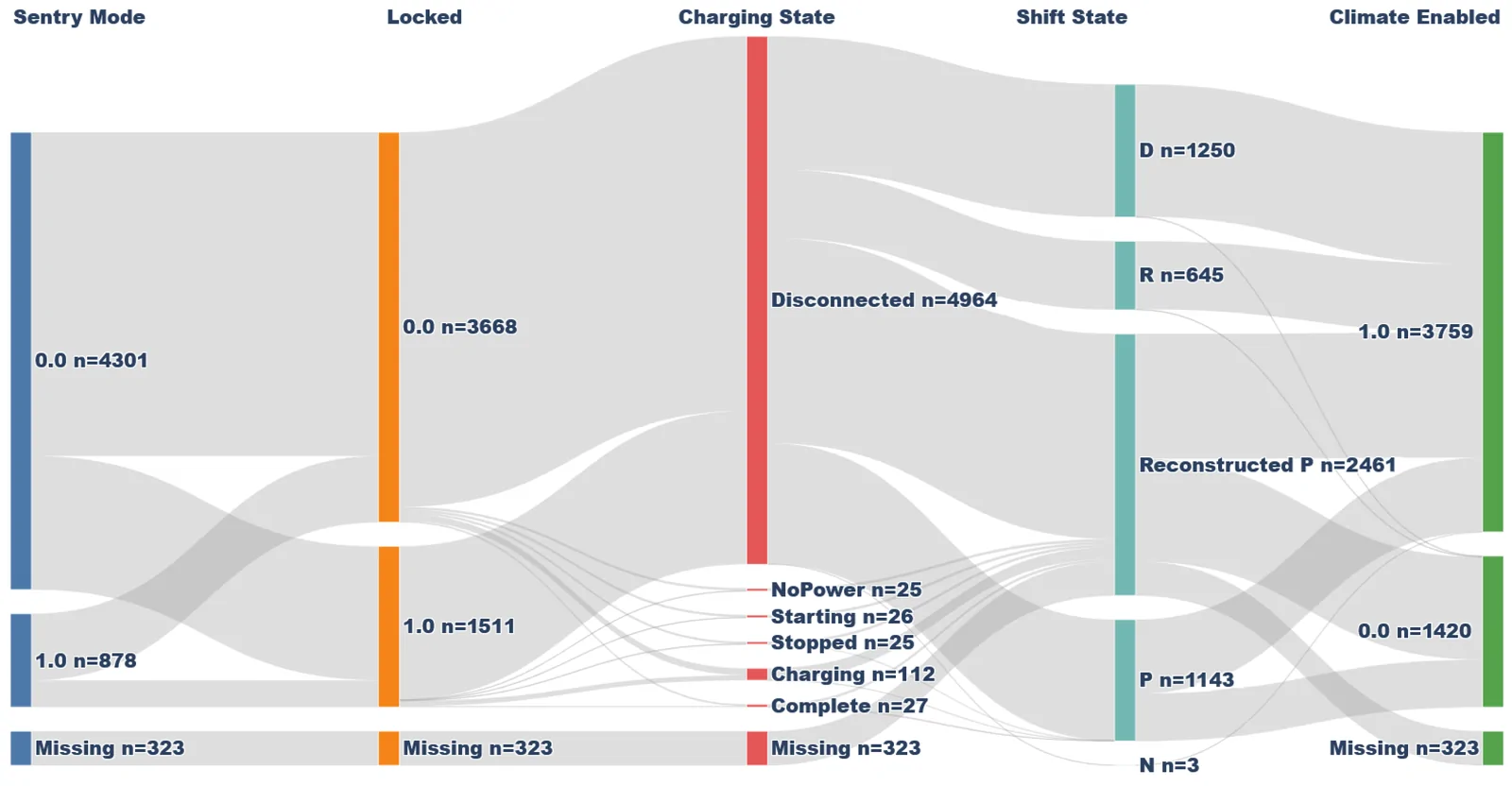
Sankey diagram of all the categorical variables available.

As a bridge between the shift states and the charging states, information on the transition and relation between the recorded states of the Tesla can be found in Figure 15, where a Sankey diagram relating all the categorical variables and the associated values can be found.

**Figure 16:**
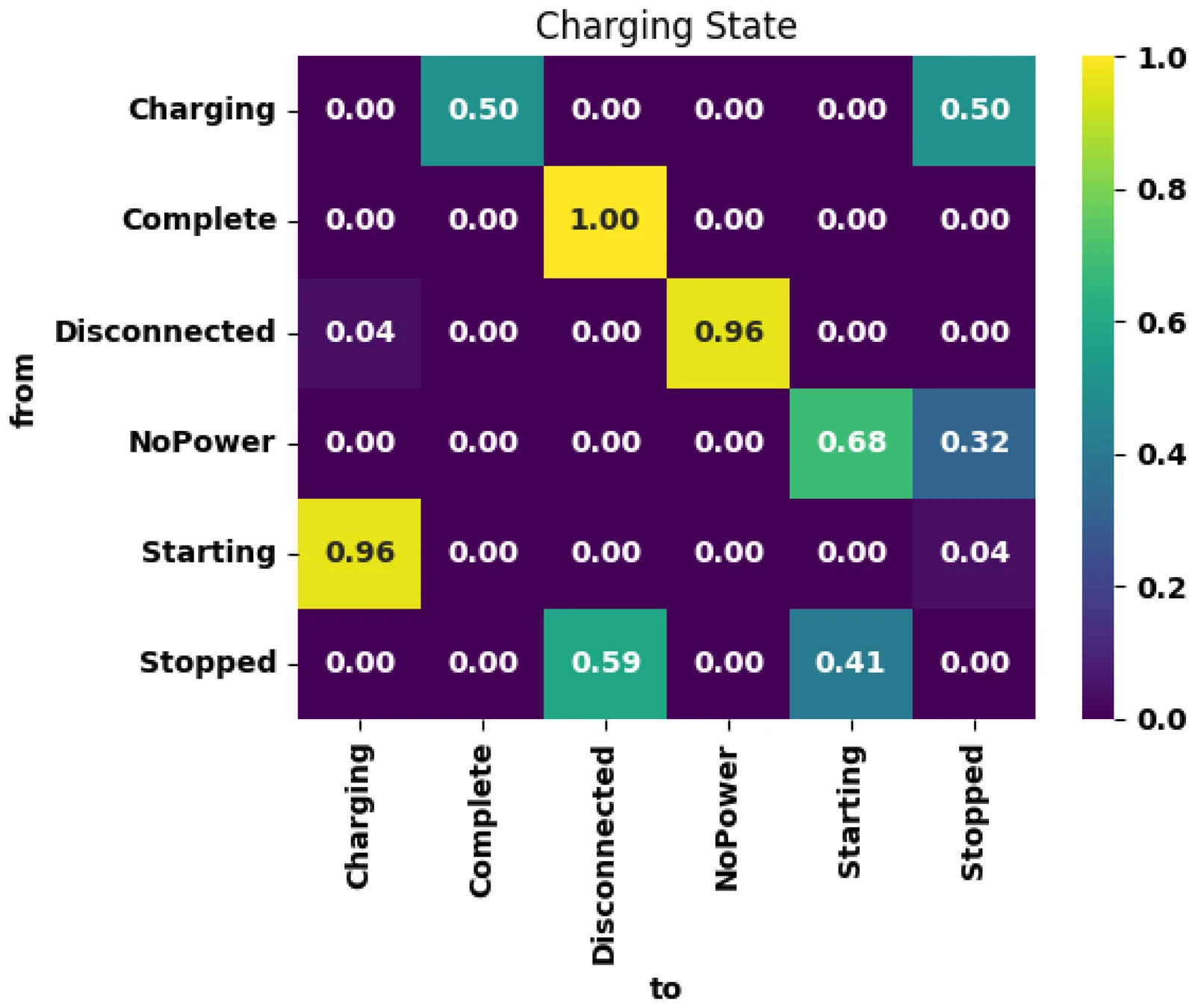
Transition matrix of the charging states.

As mentioned, in the overall dataset, actual charging constitutes a rather low percentage of the overall charging states, with the dominant category being ‘disconnected’ and a relevant share of reconstructed parking originating from this state. Most times, the vehicle enters in either driving or reversing mode. Expectedly, climate-enabled positive values are strongly tied to non-parking states. Coming now to the analysis of the charging states per se, in Figure 16, the transition matrix shows the evolution of the states in the charging process that characterized the right half of previous Figure 12a.

As expected, charging sessions evolve to either completion or a forced stop, while the states characterized by absence of power are strictly tied to initialization or stopping of the process. Having given an introductory analysis of the dataset, a detailed characterization of it will now be presented in Section 3.

## 3. Results and Discussion

**Figure 17:**
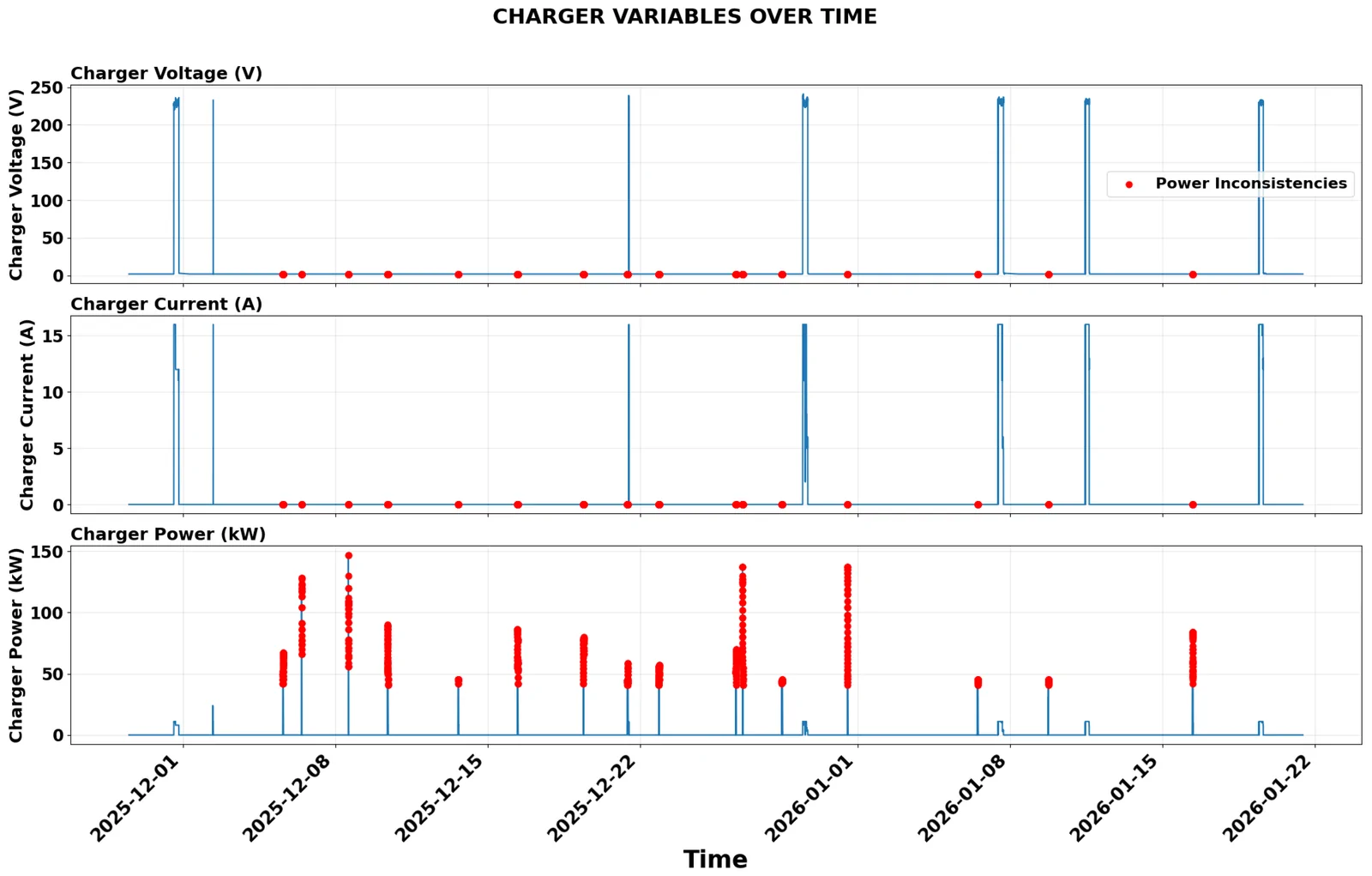
Plot of the evolution of the variables related to the charger.

Over the course of this section, the results coming from the data analysis and the complications identified will be discussed. A major identified inconsistency can be found in the charging power, reaching as high as 150 kW even at combinations of zero charging current and zero charging voltage, as visible in red in Figure 17.

**Figure 18:**
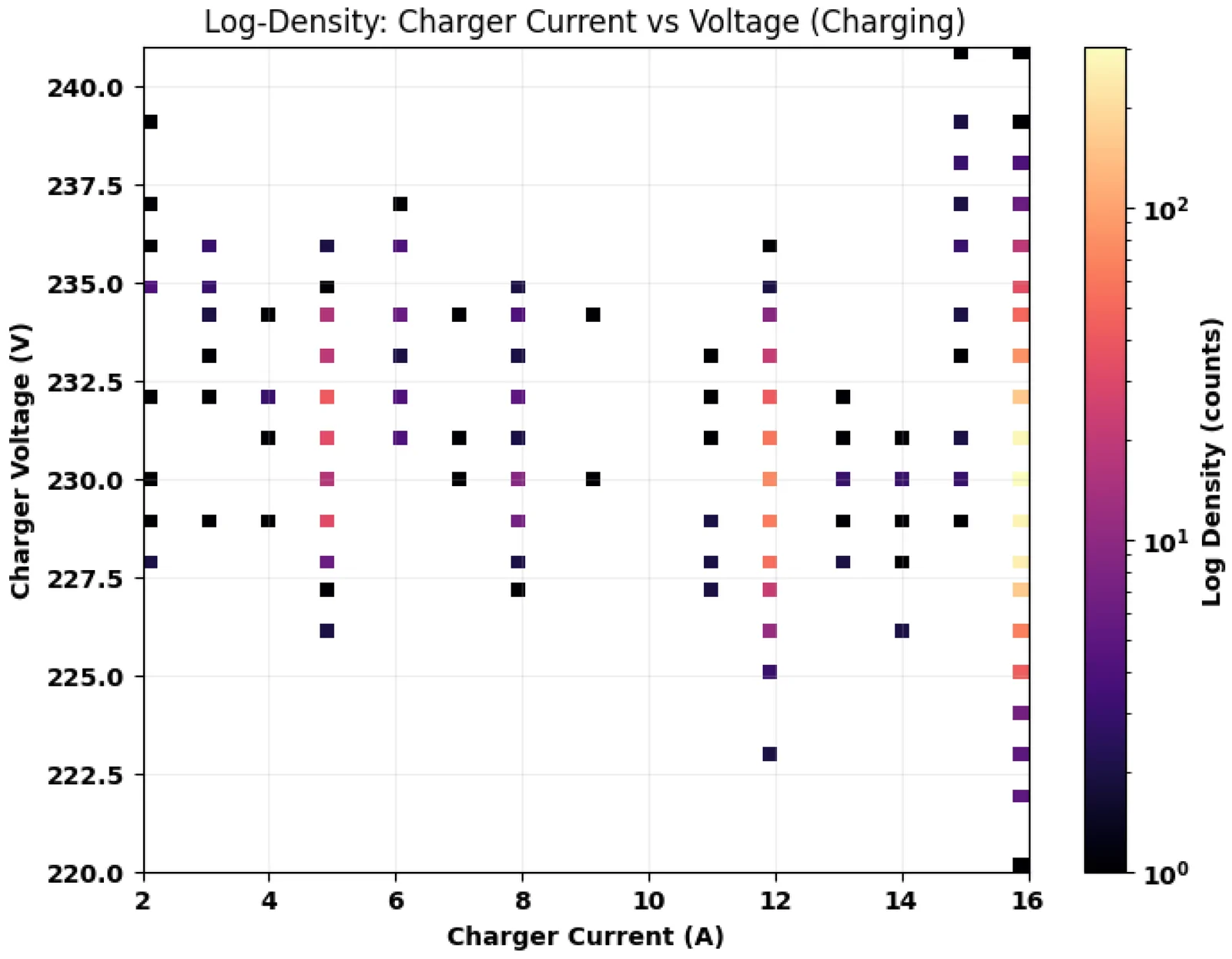
Density plot of the combination of charging voltage and current, with legend in log scale.

In multiple instances there happens to be a mismatch between the power readings of the charger and the actual power computed as the product of voltage and current, as Figure 18 depicts. As a first logical step, inspection of the combination of registered voltage and current was conducted by means of a density plot. The combination of values that presents the highest density is at (16 A, 230 V), while a particularly low density can be appreciated when current is in the range [8 A, 10 A].

**Figure 19:**
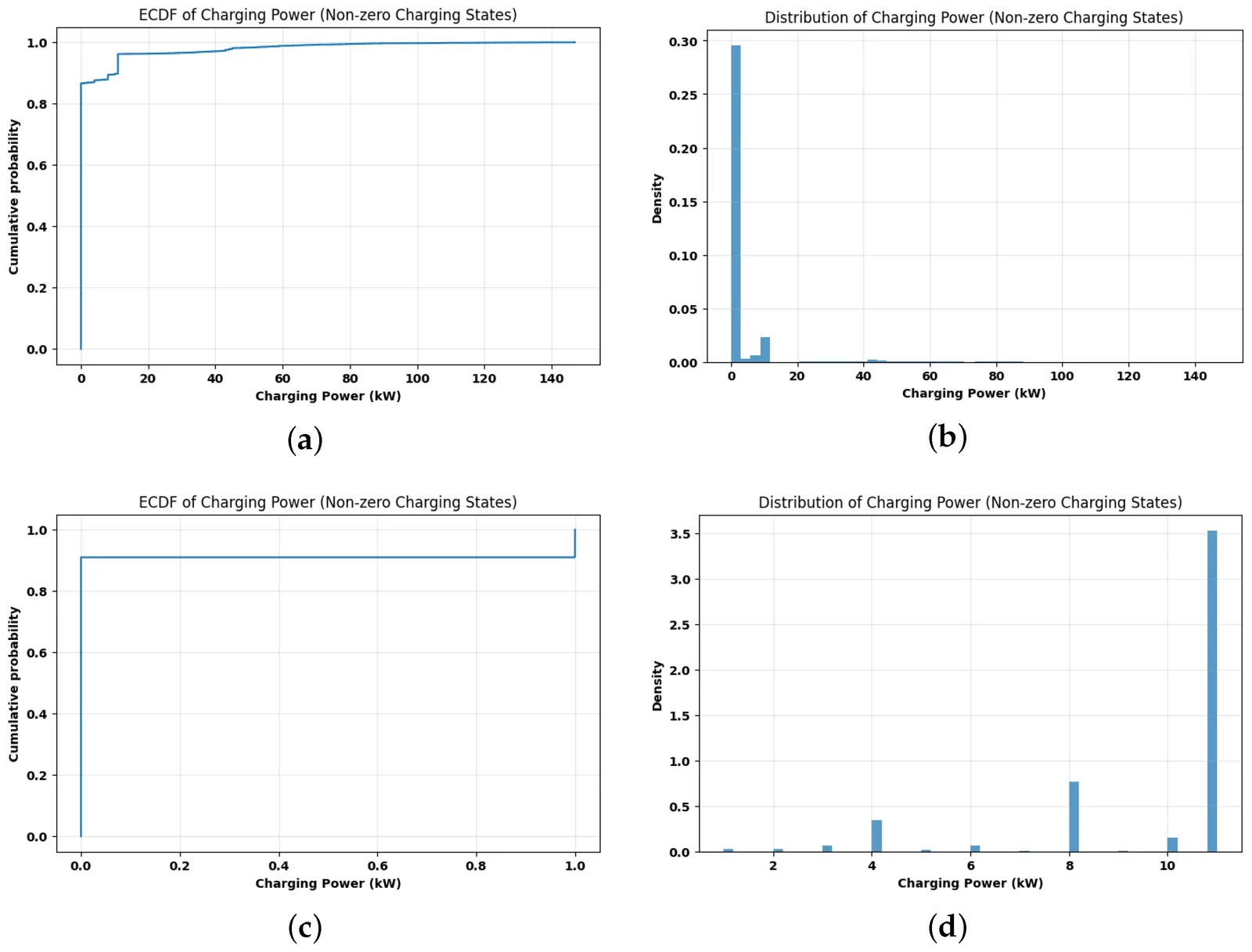
Comparison of charging power distributions under disjunctive (**a**,**b**) and conjunctive (all non-zero) (**c**,**d**) filtering conditions.

By varying the method of identification of the charging subset, importantly differing results are obtained and presented in Figure 19. In the image, with disjunctive, it is intended that at least one of the variables is non-zero (corresponding to the weak definition), while, with conjunctive, it is intended that all the variables related to charging are contemporarily non-zero. In particular, by comparing the two ECDFs, it is possible to appreciate a structurally different repartition of the values in the distribution. While both identified states present a spike in the composition for very low charging power values, the distribution contributing to shares above 90% is oriented to strongly higher values in the conjunctive charging state definition.

**Figure 20:**
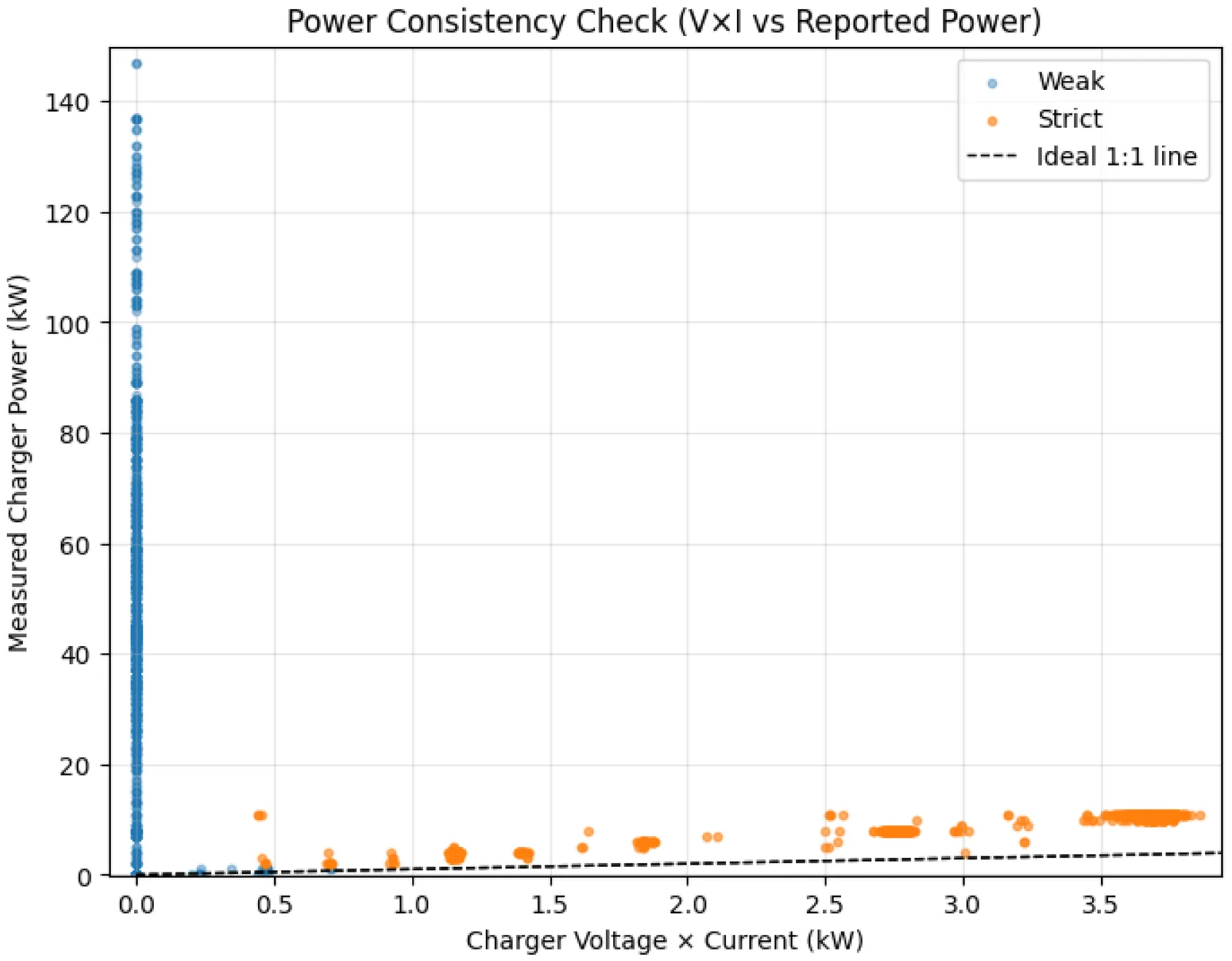
Scatter plot highlighting the instances where the registered power from the charger is consistent with the readings from charger current and charger voltage.

Such findings led the authors to pursue the definition of the set of regimes introduced in Section 2.1 and consequentially inspect the dataset under these perspectives. As a matter of fact, by visualizing in Figure 20 the product of the voltage and current during charging phases and the registered associated charging power, it is possible to identify a noticeable issue with the power and voltage/current data.

**Figure 21:**
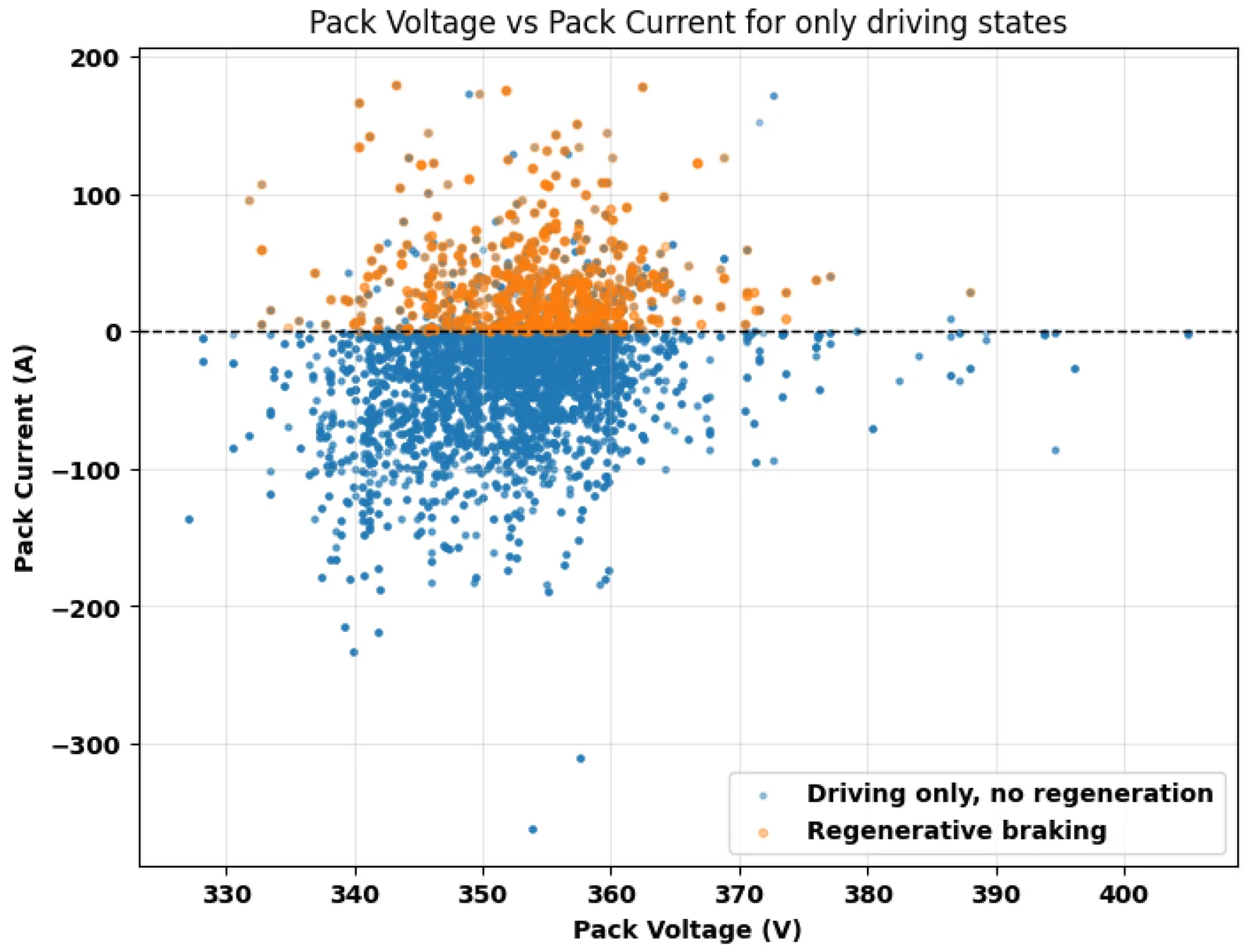
Scatter plot of the combination of pack voltage and pack current, colored based on the regenerative masks previously defined.

The differences in values seem to be correlated with the previously defined weak and strict charging masks, with computed powers above 0.5 kW pertaining to the strict definition in which all charging values must be jointly positive. Before developing an analysis of the clusters identified, an overview of the regenerative braking state is provided in the following Figure 21.

**Figure 22:**
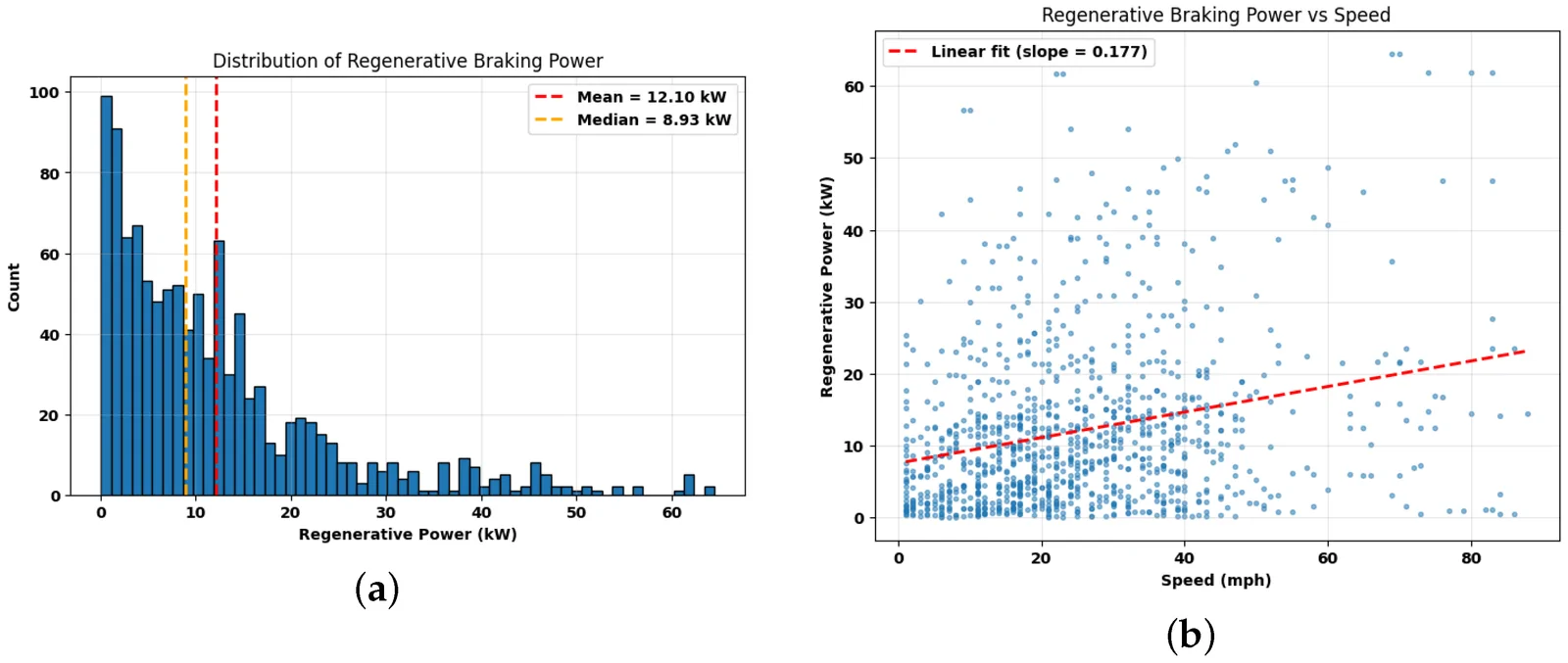
Combination of number of instances of regenerative power (**a**) and the speed at which such power is recovered (**b**).

For completeness, the blue points in the above plot that fall in the above-zero pack current sector have been inspected and are simply not pertaining to the shift state ‘D’ or further filtered out by acceleration conditions. Instances of regeneration are associated with an average value of 12.10 kW and a median of 8.93 kW, distributed in a multimodal manner with secondary peaks at 12 kW, 14 kW and 23 kW, as visible in Figure 22a.

**Figure 23:**
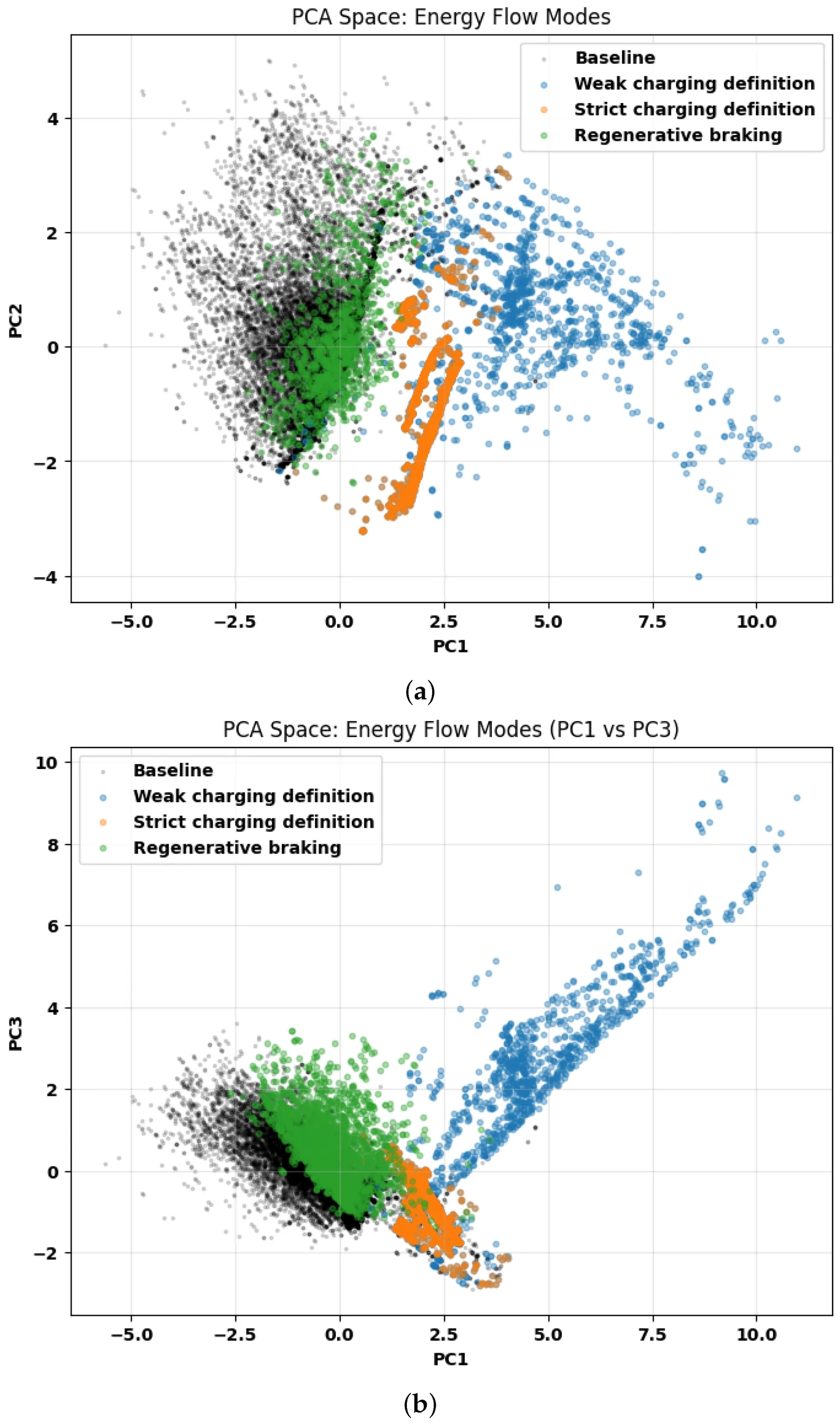
PCA results characterized by charging mask for PC1 vs. PC2 (**a**) and for PC1 vs. PC3 (**b**).

It is now possible to plot the characterizations identified of the previously computed PCA, visible in Figure 23a for PC1 vs. PC2 and Figure 23b for PC1 vs. PC3. The characterization proposed through the charging masks is able to identify four spaces and distributions of data in a two-dimensional representation, allowing for visual inspection of the dataset. The weak charging and strict charging definitions do not overlap, with the former showing a rather large dispersion, while the baseline mask and the regenerative one prove to be pertaining to the same space as expected. Interestingly, and yet expectedly, the regeneration of energy by braking brings the instances in the dataset closer to the charging states of the weak and strict masks. To provide readers with a measure of the cohesion and strength of identity of the operational regimes of the vehicle, metrics are computed using geometric statistics in the embedded feature space. Table 8 summarizes the compactness, centrality, dispersion, and Gini spread for each regime. These metrics jointly describe intra-cluster cohesion, global positioning in feature space, and internal heterogeneity.

**Figure 24:**
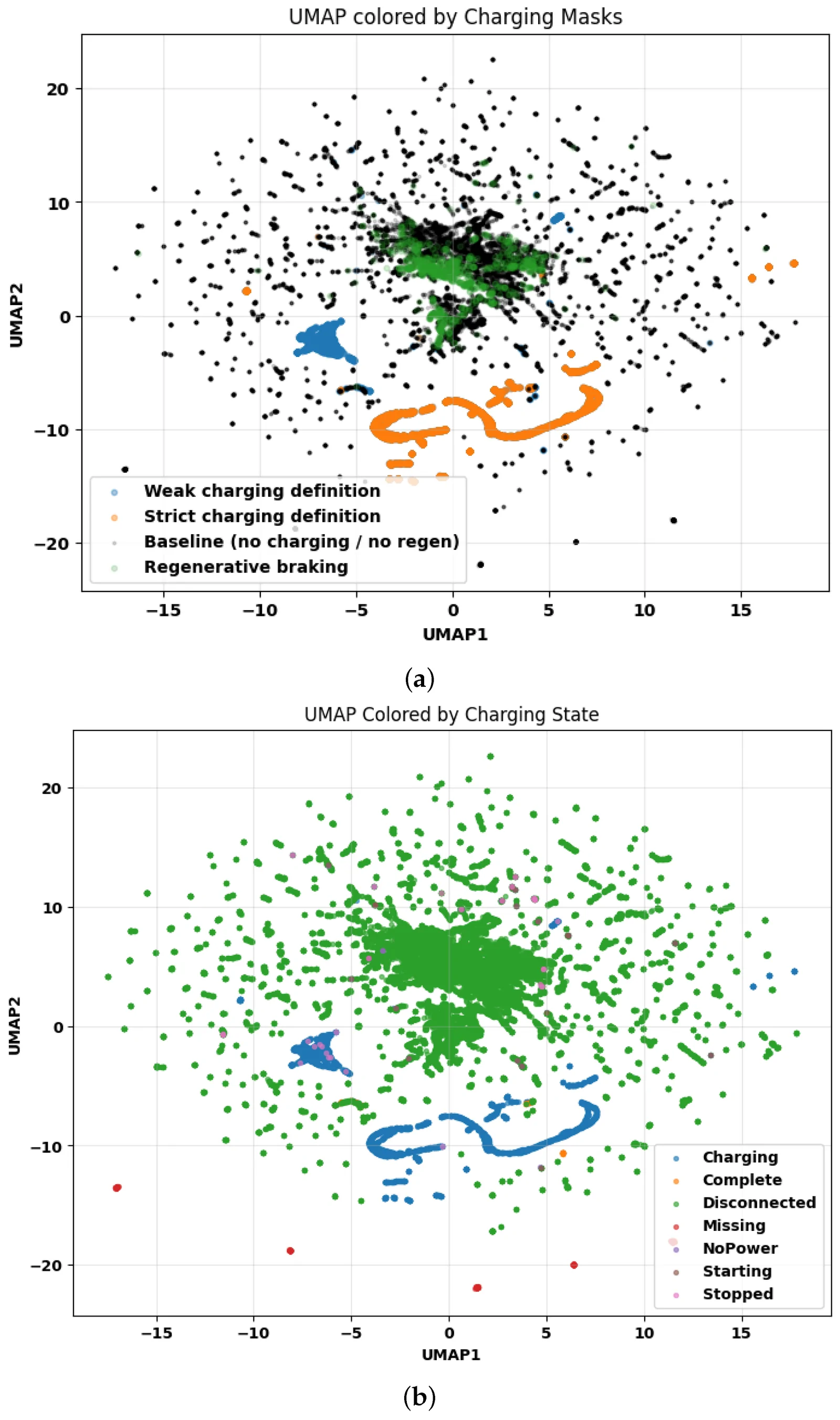
UMAP results characterized by (**a**) defined masks and (**b**) reported charging state.

Strict charging exhibits the lowest compactness (1.040) and dispersion (1.433), indicating the most constrained and internally coherent operating regime among the identified behavioral states. This compact structure reflects the strong physical constraints imposed by the charging process, where voltage–current interactions and energy transfer follow relatively stable processes. Its centrality (2.534) indicates that strict charging occupies a distinct region of the feature space while remaining connected to the broader operational manifold. The moderate Gini spread (0.323) suggests that, despite its compact structure, internal variability remains present, likely due to differences in charging conditions and the coexistence of multiple charging phases, such as constant-current and constant-voltage operation. Weak charging presents the highest compactness (2.490), dispersion (3.301), and centrality (2.957) among the charging-related regimes, indicating a broader and less constrained region in the feature space. This regime seems to be associated with transitional charging behavior, including low-power charging events, partial charging activation, transient electrical states, and marginal operating conditions. The combination of elevated dispersion and centrality suggests that weak charging does not represent a single stable operating mode but rather a heterogeneous region located away from the dominant operational manifold. Its relatively low Gini spread (0.276) indicates that variability is distributed relatively uniformly within this regime despite its larger geometric extent. Regenerative braking forms an intermediate regime, characterized by moderate compactness (1.399) and dispersion (1.975), with a centrality value (0.635) close to that of the baseline regime. This proximity indicates that regenerative braking remains embedded within the main vehicle operational manifold rather than forming a strongly isolated state. The Gini spread (0.288) indicates moderate internal variability, reflecting differences in braking intensity, recovered power levels, vehicle speed, and transient deceleration dynamics. Therefore, regenerative braking appears as a distinct but highly integrated operating condition within normal vehicle behavior. The baseline regime represents the dominant behavioral manifold of the vehicle, containing the majority of observations (21,104 samples) and exhibiting the lowest centrality (0.486). Its compactness (1.503) and dispersion (2.102) indicate moderate internal variability, which is expected given that this regime encompasses a wide range of common driving conditions, including cruising, acceleration, deceleration, and idling. Rather than representing a narrowly constrained operating state, baseline behavior defines the reference region around which specialized operational regimes are organized. Overall, the results reveal a structured geometric organization of vehicle operation. Strict charging forms the most compact and physically constrained regime, while weak charging occupies a broader transitional region associated with less stable charging conditions. Regenerative braking remains integrated within the core operational manifold, and baseline driving defines the primary behavioral attractor of the system. These findings indicate that operating regime separability is determined not only by cluster compactness but also by the relative positioning and distribution of each regime within the vehicle’s behavioral feature space. At the end of this analysis, considering that the distribution of explained variance would require a four-dimensional representation for visual inspection of the results to achieve an 84.7% total explained variance ratio, a visual validation is provided in Figure 24a,b. For all the regimes except the general baseline (in black in the images), points pertaining to different shift states in the UMAP space are closer together when compared to the PCA representation. A major difference in the discriminatory power can be found in the weak charging definition, for which UMAP seems to be able to identify higher coherence and similarity between the entries than PCA, while the opposite can be appreciated for the strict charging definition. The main reasons associated with these findings are that:

1. UMAP is able to identify nonlinear relationships among data, with increased attention towards closer instances.
2. PCA not only overlooks single-point characteristics in favor of targeting the variance of the overall dataset but also achieves this from a linear perspective, possibly dampening the peculiar relationships among close same-regime entries.

The above reasons might then play a role in the representation of the points in the respective embedded spaces, especially with respect to the inconsistencies in the charger’s power and voltage records. Independently from the visualization chosen, and thus the relative importance attributed to global or local structures, both representation methods can be considered valid and lead to the same conclusions.

## 4. Conclusions

This work presented a critical analysis of a real-world dataset collected from a Tesla Model 3 operating under everyday driving conditions in northern Italy. The study was motivated by the growing role of data-driven methodologies within the Industry 4.0 and Industry 5.0 paradigms, where digital twins are increasingly adopted to support monitoring, prediction, and decision-making processes in complex systems such as electric vehicles. The analysis focused on assessing the plausibility and consistency of the available variables with the objective of understanding whether the dataset could constitute a reliable validation basis for a corresponding digital twin and virtual reality driving simulator developed in related works. To this end, exploratory analyses, operational regime classification, dimensionality reduction techniques, and feature-space characterization were employed to investigate the structure and coherence of the recorded information. The results highlight that the dataset contains a substantial amount of information that is capable of describing the vehicle’s operational behavior under different driving and charging conditions. Distinct operating regimes, including charging, regenerative braking, and baseline driving, could be identified and characterized through their geometric properties in the latent feature space. At the same time, several limitations emerged, including missing variables, ambiguities in the interpretation of some signals, and the absence of direct measurements for a number of quantities required by high-fidelity physical models. Among the outstanding information identified, there is a lack of coherence between the registered charger power and the charger’s current and voltage. Comparing the available information on the delivered power and its effect on the SOC of the vehicle, a plausible cause can be identified in a miscommunication between the chargers and the vehicle, possibly due to different communication protocols. This hypothesis is further supported by the single location associated with the charging sessions, corresponding to the vehicle owner’s location and thus lacking public charging sessions. These limitations do not prevent the use of the dataset for validation purposes, but they constrain the level of detail and physical accuracy that can be achieved by the associated digital twin. Additionally, one of the main limitations of the analysis can be identified in the lack of variability in the driver’s inputs. In other words, since the data has been collected from a single vehicle and a single driver, a number of behaviors, and, in turn, corresponding operating conditions, could be under-represented and under-analyzed. Although this limitation has to be acknowledged and considered while further analyzing the data, it is also partially attenuated by the mixture of urban and extra-urban driving locations, as well as the rather vast temperature range across time. Furthermore, the method described in this study lacks predictive modeling and classification, avoiding misrepresentation of unprecedented data. From a broader perspective, the findings confirm that the quality of the data acquisition chain remains a critical factor for the successful implementation of digital twins in mobility applications. Even when large quantities of telemetry are available, issues related to sensor selection, signal reliability, sampling strategies, and data completeness can significantly influence the validity of the resulting models. Consequently, systematic data validation should be considered as a prerequisite for the development of digital twins that are intended for predictive analyses and human-in-the-loop simulations. Future work should focus on extending the dataset with additional vehicle, environmental, and driver-related measurements while also improving traceability and synchronization among data sources. Additionally, the possibility of developing automatic outlier detection systems, statistics flagging and comparison, and, in general, classification models will enhance the ability of the collected data to be automatically scrutinized and fed to the digital twin. Such developments would enable more accurate model calibration and strengthen the correspondence between real-world vehicle behavior and its virtual counterpart, ultimately contributing to the realization of more reliable and effective digital twin frameworks for electric vehicles.

## Figures and Tables

**Table 1:** Classification of EV performance evaluation models.

Model Type	Scope	Typical Inputs	Main Applications
Analytical/ Mathematical	First-order vehicle energy estimation	Vehicle mass, speed, drag coefficient, road slope	Preliminary design and rapid assessment
Component-Based Physical	Battery, motor, inverter and drivetrain representation	Electrical, thermal and mechanical parameters	Component design, optimization and validation
Driving Simulators	Vehicle performance with driver interaction	Driver inputs, traffic conditions, road geometry	Eco-driving and human factor studies
Digital Twins	Real-time virtual representation of a physical vehicle	Telemetry, sensor data, operational states	Predictive maintenance and fleet management
Machine Learning	Data-driven prediction and inference	Historical telemetry, environmental and operational data	Predictive analytics and intelligent energy management
Telemetry-Based Monitoring	Analysis of real-world vehicle operation	CAN bus data, GPS and other measurements	Fleet monitoring and validation studies
Fleet-Level Models	Aggregated behavior of multiple EVs	Fleet telemetry, charging demand, mobility patterns	Charging infrastructure planning, smart charging and V2G studies

**Table 2:** Tesla Model 3 technical characteristics.

Parameter	SI Unit	Value
Dimensions (L × W × H)	[m]	4.72 × 2.89 × 1.44
Curb weight	[kg]	1655
Maximum speed	[km/h]	225
Battery voltage	[V]	359
Motor rated power	[kW]	202
Consumption	[kWh/100 km]	13.0
Range (WLTP)	[km]	534

**Table 3:** Missingness categories across the synchronized dataset.

Variables	Missing Data (%)
Front/rear tire pressure sensors	99.6
Latitude, longitude, elevation, speed, odometer, shift state	49.2
Battery, charging, temperature, locking, and power-related variables	1.54

**Table 4:** Battery statistics across shift states.

Variable	All	Drive	Parking	Reconstructed Parking
Energy Remaining (kWh)	37.38 ± 13.40	37.41 ± 13.34	37.65 ± 14.16	37.37 ± 13.30
Pack Current (A)	−1.92 ± 45.55	−20.08 ± 45.72	−6.74 ± 26.78	14.37 ± 42.11
Pack Voltage (V)	356.14 ± 7.88	353.20 ± 7.20	354.44 ± 6.93	358.93 ± 7.58
Min Battery Module Temperature (°C)	16.91 ± 8.59	16.66 ± 8.32	14.85 ± 7.06	17.46 ± 8.98
Max Battery Module Temperature (°C)	18.03 ± 9.40	17.93 ± 9.25	15.87 ± 7.80	18.48 ± 9.70
Usable Battery Level (%)	61.98 ± 24.27	62.07 ± 23.97	62.41 ± 25.35	61.95 ± 24.32
Battery Range (mi)	163.03 ± 63.93	163.31 ± 63.27	164.34 ± 66.92	162.90 ± 63.95
Charger Current (A)	1.27 ± 4.19	0.00 ± 0.03	0.00 ± 0.00	2.59 ± 5.68
Charger Power (kW)	2.95 ± 11.64	0.00 ± 0.00	0.04 ± 1.34	6.00 ± 16.04
Charger Voltage (V)	22.73 ± 65.52	2.05 ± 2.91	2.01 ± 0.12	44.11 ± 88.45

**Table 5:** Variance inflation factor (VIF) of the variables retained for PCA.

Variable	VIF
Pack Voltage (V)	11.81
Energy Remaining (kWh)	10.33
Speed (mph)	3.57
Battery Temperature Spread (°C)	2.88
Charger Power (kW)	2.38
Pack Current (A)	2.26
Charger Current (A)	1.17

**Table 6:** Explained variance ratio of the first four principal components.

Principal Component	Explained Variance Ratio
PC1	0.335
PC2	0.209
PC3	0.164
PC4	0.138

**Table 7:** PCA loadings for the reduced variable set.

Variable	PC1	PC2	PC3
Energy Remaining (kWh)	0.241773	0.536691	−0.505295
Pack Current (A)	0.512555	−0.307355	0.163808
Pack Voltage (V)	0.530343	0.165315	−0.303751
Battery Temperature Spread (°C)	0.246646	−0.247600	−0.022276
Charger Current (A)	0.499437	−0.126863	0.452589
Charger Power (kW)	−0.170961	0.429815	0.567067
Speed (mph)	0.240978	0.572735	0.314135

**Table 8:** Geometric statistics of operational regimes in embedded feature space. Lower compactness and dispersion indicate more structured regimes, while centrality measures the distance from the global behavioral manifold center. Gini spread quantifies internal heterogeneity within each regime.

Group	No. of Samples	Compactness	Centrality	Dispersion	Gini
Weak Charge	3591	2.490	2.957	3.301	0.276
Strict Charge	2423	1.040	2.534	1.433	0.323
Regenerative	2252	1.399	0.635	1.975	0.288
Baseline	21,104	1.503	0.486	2.102	0.295

**Figure A1:**
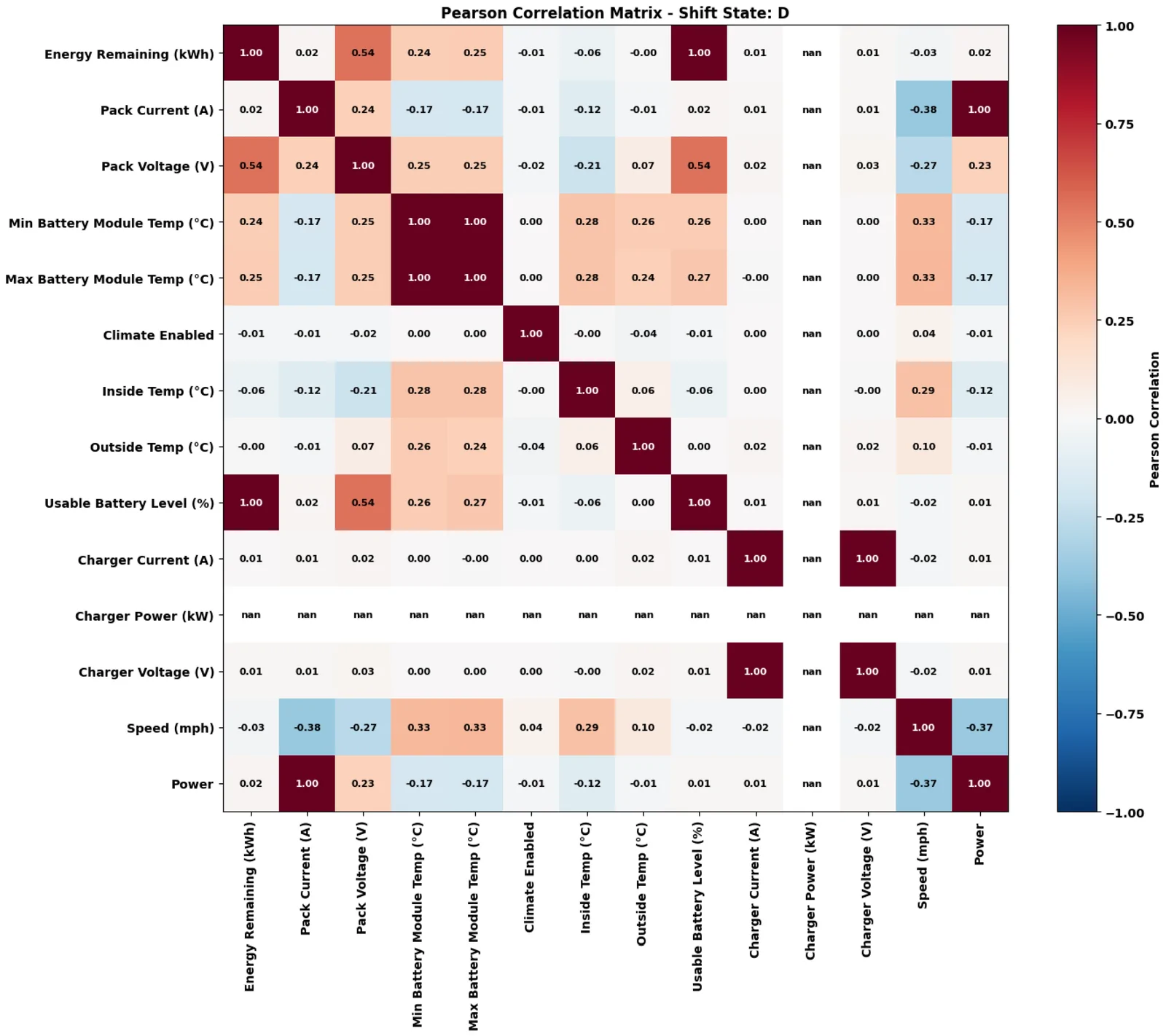
Pearson’s correlation computed for points belonging to shift state of driving.

**Figure A2:**
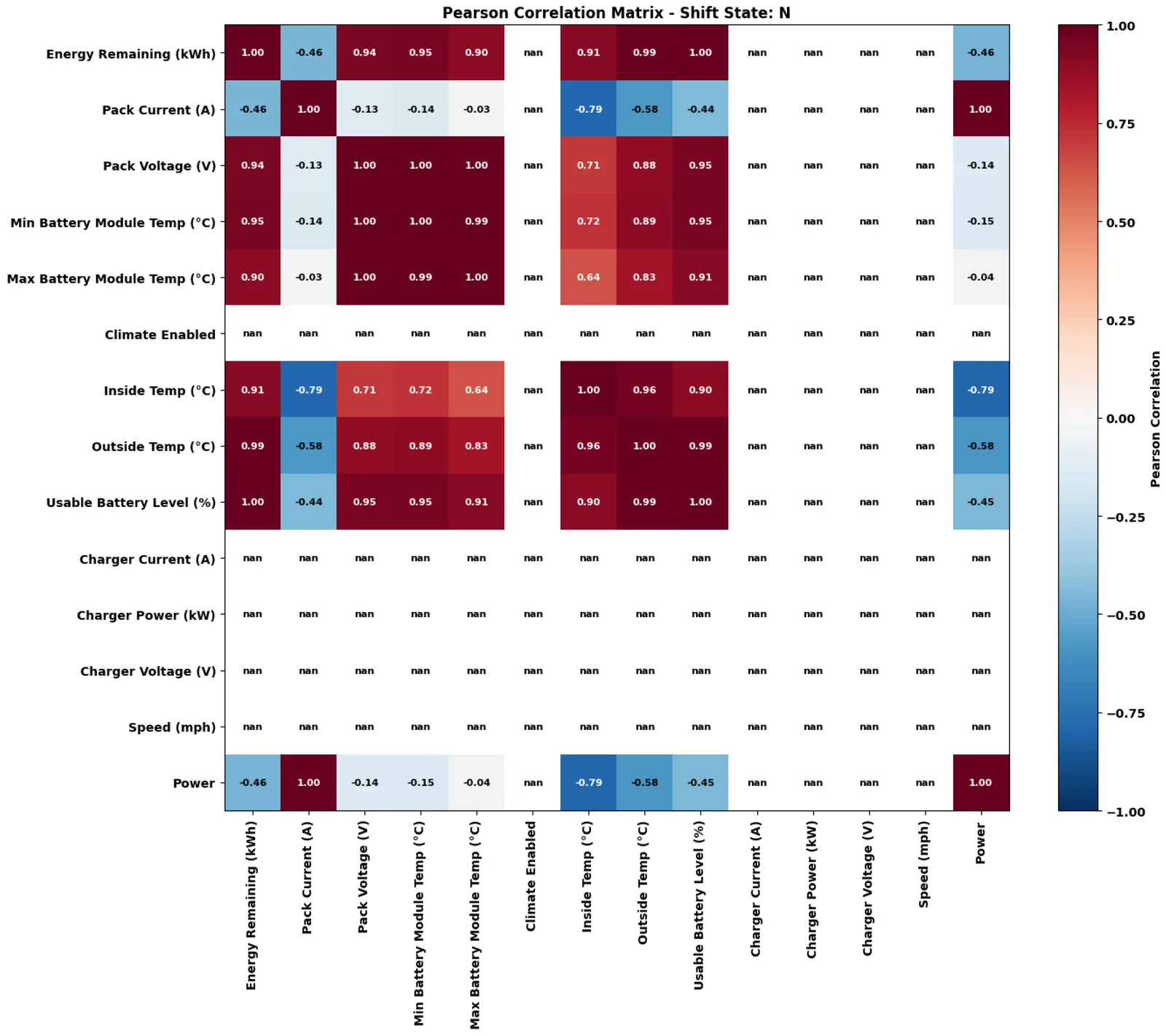
Pearson’s correlation computed for points belonging to shift state of neutral.

**Figure A3:**
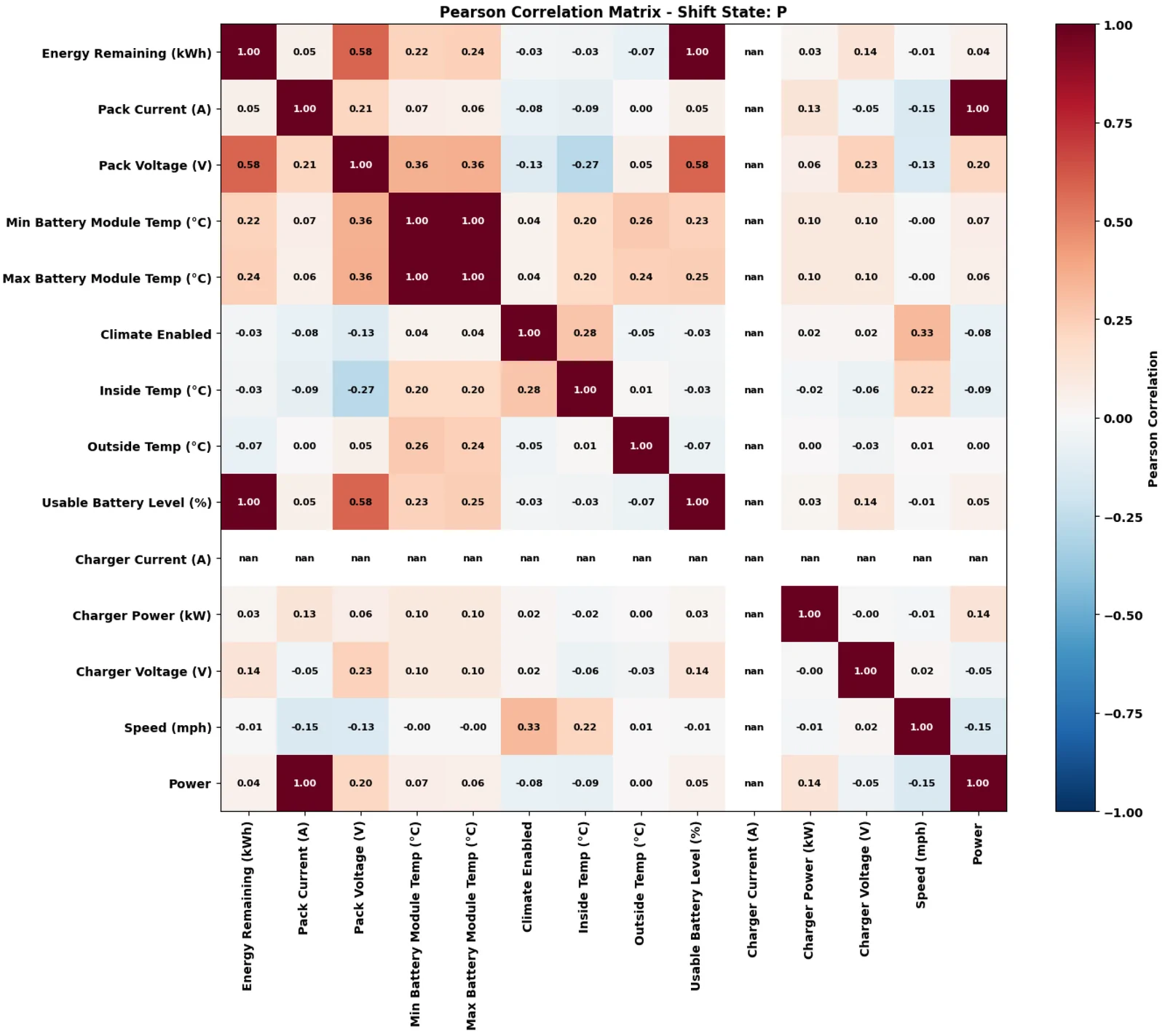
Pearson’s correlation computed for points belonging to shift state of parking.

**Figure A4:**
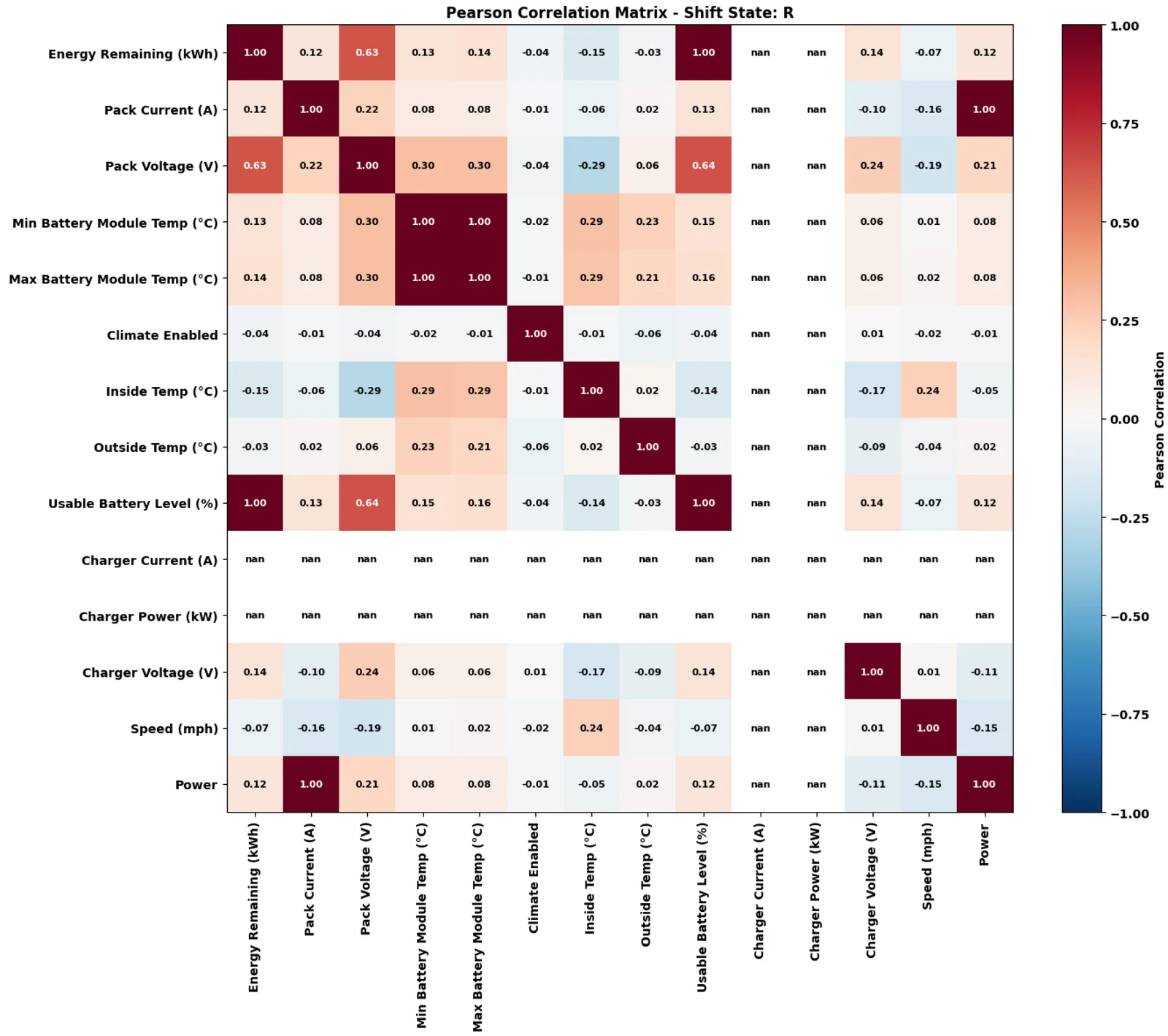
Pearson’s correlation computed for points belonging to shift state of reversing.

## Data Availability

The raw data supporting the conclusions of this article will be made available by the authors on request.

## Appendix A

As mentioned in Section 2.2, shift-segmented Pearson’s correlations are presented in the following.
